# How do people distribute their attention while observing *The Night Watch*?

**DOI:** 10.1177/03010066221122697

**Published:** 2022-09-29

**Authors:** Joost C. F. de Winter, Dimitra Dodou, Wilbert Tabone

**Affiliations:** 2860 Delft University of Technology, The Netherlands

**Keywords:** eye-tracking, painting, Rembrandt, salience, texture

## Abstract

This study explored how people look at *The Night Watch* (1642), Rembrandt's masterpiece. Twenty-one participants each stood in front of the painting for 5 min, while their eyes were recorded with a mobile eye-tracker and their thoughts were verbalized with a think-aloud method. We computed a heatmap of the participants’ attentional distribution using a novel markerless mapping method. The results showed that the participants’ attention was mainly directed at the faces of the two central figures, the bright mascot girl in the painting, and detailed elements such as the apparel of the key figures. The eye-movement analysis and think-aloud data also showed that participants’ attention shifted from the faces of the key figures to other elements of the scene over the course of the 5 min. Our analyses are consistent with the theory that Rembrandt used light and texture to capture the viewer's attention. Finally, the robustness of the eye-tracking method was demonstrated by replicating the study on a smaller replica.

What do people look at in Rembrandt van Rijn's *Militia Company of District II under the Command of Captain Frans Banninck Cocq*, more commonly known as *The Night Watch*? This question has remained unanswered to date, presumably for two reasons. Firstly, *The Night Watch* is not easily accessible for research with human participants. *The Night Watch* is one of the most precious paintings globally, and conducting secure research on this painting involves considerable logistics and preparation. Secondly, it is challenging to map the eye movements, measured with a mobile eye-tracker, to a frontal image of a large painting.

Eye-tracking research on real artworks is rare (for exceptions, see e.g., Balbi et al., 2016; Brieber et al., 2014; Quian Quiroga et al., 2011; Walker et al., 2017). Walker et al. (2017) obtained eye-movement heatmaps using a mobile eye-tracker for paintings in the Van Gogh Museum. The heatmaps were acquired with the help of 10 × 10 cm markers placed on the wall, near the corners of the paintings. Using eye-tracking software (Kassner et al., 2014), the video footage of the mobile eye-tracker was mapped to a frontal image of the painting. The study by Walker et al. was conducted on five paintings, the smallest having a length × width of 46 × 39 cm and the largest 101 × 50 cm, viewed from a relatively large distance of 3 m. More recently, Fontoura and Menu (2021) created a heatmap for a 141 × 269 cm painting viewed by visitors of the Unterlinden Museum in France. They used the eye-tracker developer's Real-World Mapping algorithm to map gaze data to snapshots of the painting without using markers. The method appeared to yield accurate heatmaps, although 10% of the mappings had to be performed manually. Similar methods in real museums were used by Estrada-Gonzalez et al. (2020), Grazioso et al. (2020), and Mandolesi et al. (2022). However, the above methods may not be optimal to be applied to *The Night Watch*, a painting that measures 437 × 363 cm and features large dark regions. In this study, we developed a custom mapping method for obtaining heatmaps.

Rembrandt, sometimes referred to as the “*master of light and shadow*” (e.g., Weiss, 1996, p. 17), is known for modulating the amount of detail and light in his paintings. Warshow (2014) noted about *The Night Watch*: “*what sets Rembrandt's group portrait apart from other comparable paintings is his use of chiaroscuro as a dramatic device*”. Haverkamp-Begemann (1982) commented on the details of the men in front in *The Night Watch*: “*Rembrandt's detailed rendering of the costume and of the partisan* [of lieutenant Van Ruytenburgh, the central figure in white]” (p. 76), while noting the absence of details elsewhere: “*In contrast to his colleagues, Rembrandt sacrificed colors and details of costumes and apparel of many of the sitters and of the background to that integration by submerging them in deep shadows and dark tones.*” (p. 69). Similar observations were made by Bruyn et al. (1989): “*In the figures of Captain Banning Cocq and Lieutenant Van Ruytenburgh … the treatment reaches its greatest intensity in terms of chiaroscuro contrast (between the two of them and within the figures themselves), sharpness and wealth of detail*” (p. 441).

Until now, the vast majority of research into eye movements when viewing paintings has been conducted using paintings displayed on a computer screen (e.g., Bauer & Schwan, 2018; Goller et al., 2019; Kapoula et al., 2009; Kirtley, 2018; for an overview, see Garbutt et al., 2020). Although these methods may lack realism compared to the experience of watching a painting in a museum or gallery, an advantage of eye-tracking research on a computer screen is that the painting can be manipulated in a controlled manner. This possibility was used by DiPaola (2008) and DiPaola et al. (2010, 2013) to examine eye movements while viewing (self-)portraits by Rembrandt. More specifically, DiPaola et al. presented images of Rembrandt's paintings and photoshopped control images that varied in the amount of texture used. Accordingly, they found support for the selective-detail hypothesis, which says that Rembrandt painted elements in greater detail to attract the viewer's attention. Similar conclusions about textural details were reached by Latif et al. (2014), who also employed eye-tracking on a computer screen. These authors concluded that textures attract the viewer's eyes. However, whether Rembrandt succeeded in capturing the viewer's attention remained to be examined for real paintings.

It is possible to quantify the level of detail of *The Night Watch* by calculating the so-called local entropy. The local entropy of a pixel is an index of similarity with pixels in its close vicinity; if many of the neighboring pixels have the same brightness, such as in the case of a uniform surface, then entropy will be low. If, on the other hand, neighboring pixels have different brightness levels, such as in the case of highly textured or detailed portions, then entropy will be high. From an inspection of the entropy of the entire painting (Figure 1), it is evident that the suit of lieutenant Van Ruytenburgh, that is, the central figure in white, contains a substantial amount of detail. On the other hand, the attire of captain Banninck Cocq, that is, the central figure in black, contains little detail except for his collar. It is also striking that the girl and some of the militants’ faces have ample detail. The findings shown in Figure 1, together with the observations of DiPaola et al. (2013), allow us to hypothesize which facets of the painting will attract the most attention.

**Figure 1.**
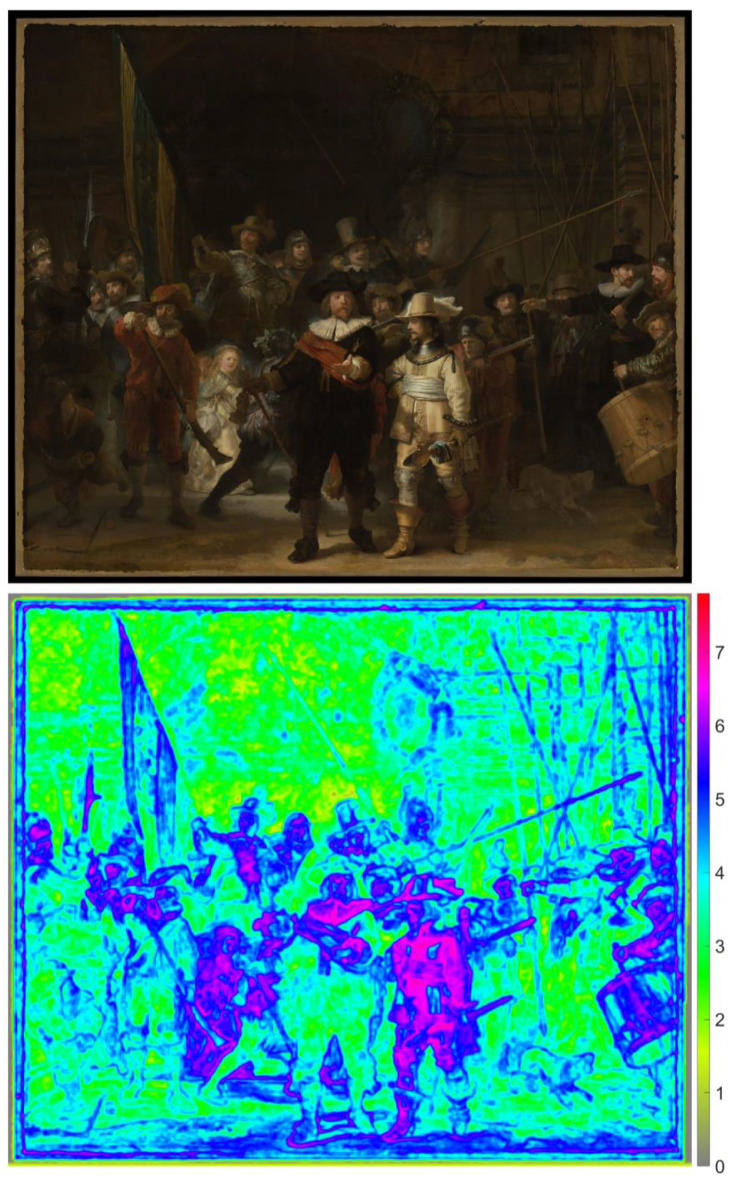
Top: Image of *The Night Watch* (2444 × 2048 pixels) (Rijksmuseum, 2020). Bottom: Saliency map consisting of the local entropy computed for the upper image in grayscale. The entropy value of each pixel was calculated based on its 15-by-15-pixel neighborhood. Entropy is defined as −Σ(*p* × log_2_(*p*)), where *p* contains the normalized histogram counts in 256 bins. The minimum possible entropy value is 0, which would occur when all 225 (15 × 15) pixels have the same grayscale value. The maximum possible entropy value is 7.81, which would occur when all pixels have a different grayscale value. The maximum entropy in this image is 7.05.

In this study, we had participants look at *The Night Watch* to discover how they distributed their attention over the painting. In addition to measuring eye movements, we used a think-aloud method to assess whether patterns in eye movements were associated with self-reported observations. The experiment was surrounded by a television crew, and this research received brief attention (3 min) in the Dutch television program *The Master's Secret* (AVROTROS, 2022). The experiment was repeated with a smaller-scale replica of the painting to determine the robustness of our eye-tracking method.

## Methods

### Participants

The participants were recruited by contacting acquaintances of the authors and television crew, and by inviting (ex-)students who lived in reasonable proximity to the Amsterdam Rijksmuseum. The invitation asked participants not to wear spectacles or wear contact lenses instead, as the use of spectacles in combination with eye-tracking is not possible. The invitation also asked participants to indicate if they would see poorly at a 5 m distance (no one replied to this query). Finally, participants were required to be able to speak Dutch.

A total of 21 persons (11 female, 10 male) participated in the experiment over two separate days (2 June 2021, *n* = 16; 11 June 2021, *n* = 5). The participants were on average 40.5 years old (range: 15–77 years, *SD* = 19.0). More details about the participants are provided in Table 1. Half of the participants owned a so-called Museum Pass, a national pass that gives access to many museums in the Netherlands; this is considerably higher than the national average of 8% (Dutch Museum Association, 2021). The responses to the other items also suggest that, although participants were not art experts, many of them had an interest in art. Participants provided written informed consent, including parental consent where appropriate. The research was approved by the Human Research Ethics Committee of the TU Delft. Participants were offered a reimbursement of their travel costs.

**Table 1.** Responses to the post-experimental questionnaire for the real *Night Watch* experiment (number of participants and percentages).

**Question**	**Response options**				
	**No**	**Yes**			
Q1. As an adult, I took classes about art.	18	3			
	86%	14%			
Q2. I took part in education about art.	18	3			
	86%	14%			
Q3. I have a Museum Pass.	10	11			
	48%	52%			
	**0**	**1–3**	**4–6**	**7–9**	**10 or more**
Q4. On average, how many times a year do you visit an art exhibition, gallery, or museum (before COVID-19)?	0	10	6	2	3
	0%	48%	29%	10%	14%
	**0**	**1**	**2**	**3**	**4 or more**
Q5. On average, how many hours a week do you create visual art?	13	3	0	0	4
	65%	15%	0%	0%	20%
Q6. On average, how many hours a week do you read about art?	10	7	3	0	1
	48%	33%	14%	0%	5%
Q7. How many times in total have you been to the Rijksmuseum before? (give an estimate)	Mean = 8.1, *SD* = 12.6, Median = 2, *n* = 21				
	**Not familiar at all**	**Somewhat familiar**	**Moderately familiar**	**Very familiar**	**Extremely familiar**
Q8. How familiar are you with history of painting?	4	10	7	0	0
	19%	48%	33%	0%	0%
Q9. How familiar are you with the psychology of eye movements?	12	7	1	1	0
	57%	33%	5%	5%	0%
Q10. How familiar are you with the painting techniques used in *The Night Watch*?	11	8	2	0	0
	52%	38%	10%	0%	0%
	**Strongly disagree**	**Disagree**	**Neither disagree nor agree**	**Agree**	**Strongly agree**
Q11. Before I arrived at the Rijksmuseum, I already suspected that I would look at *The Night Watch* during the experiment.	8	3	5	3	2
	38%	14%	24%	14%	10%
Q12. I have been thinking about *The Night Watch* during the past week.	6	8	0	7	0
	29%	38%	0%	33%	0%
Q13. In the past week, I have looked up information or read about *The Night Watch*.	16	3	1	1	0
	76%	14%	5%	5%	0%

### Apparatus

The experiment used Tobii Glasses 2 (firmware v1.25.6-citronkola-0; head unit 0.0.62) eye-tracker, set to 100 Hz recording with the Gaze Spot Meter setting turned off. The eye-tracker recorded a forward-facing view at 25 Hz and recorded the participant's verbal statements using an integrated microphone. A backup microphone (Olympus VP-20) was placed near the participant.

The illuminance where the participants were standing was 25–30 lx, measured with a Konica Minolta T-10MA oriented towards the painting. The illuminance with the sensor pointing towards the ceiling (having uniform lighting combined with spotlights) was about 110 lx. The post-processing computations were performed on a PC with Intel(R) Core(TM) i9-10900X CPU, 32 GB RAM, and NVIDIA GeForce RTX 3080 graphics card.

### Procedure

Participants were sent the consent form via email several days before the experiment. The form mentioned that the study aimed to investigate gaze patterns while viewing paintings. It also stated that participants would stand in front of a painting for 5 min, look at the painting, and express their thoughts. However, it was not mentioned which painting they would be looking at.

Participants arrived via an elevator in a hall adjacent to *The Night Watch*. After arrival, participants signed the informed consent form and were provided with the following instructions in written form (translated from Dutch):

Please walk behind the researcher. Keep looking at the floor and do not look at the painting. Look at the painting when the researcher says ‘start’. You will view the painting for 5 minutes. Look freely and as you please, but stay where you are. Think out loud while watching. Try to name everything that is on your mind. State not only what you are looking at but also your thoughts or feelings. Try to keep talking. Pretend that the others are not there. The researcher will let you know when the 5 minutes are up.

The decision to adopt a fixed 5-min viewing time, as opposed to self-paced viewing (cf. Brieber et al., 2014; Carbon, 2017; Reitstätter et al., 2020; Smith et al., 2017), was based on various considerations, including a previous pilot test in the Rijksmuseum and two pilot tests in other locations in the Netherlands where real-size versions of *The Night Watch* were available. In particular, it was regarded as important that all participants viewed *The Night Watch* for the same amount of time, as this would allow the construction of heatmaps and time-based analyses of viewing behavior. Additionally, in the pilot tests, participants were found to remain attentive for the entire 5-min period. Accordingly, 5 min was regarded as reasonable for participants and practically feasible in the time slots in which the experiment had to be performed. Participants came to the Rijksmuseum only for this experiment, and had to leave immediately as the museum was otherwise closed to the public because of COVID-19 measures.

**Figure 2.**
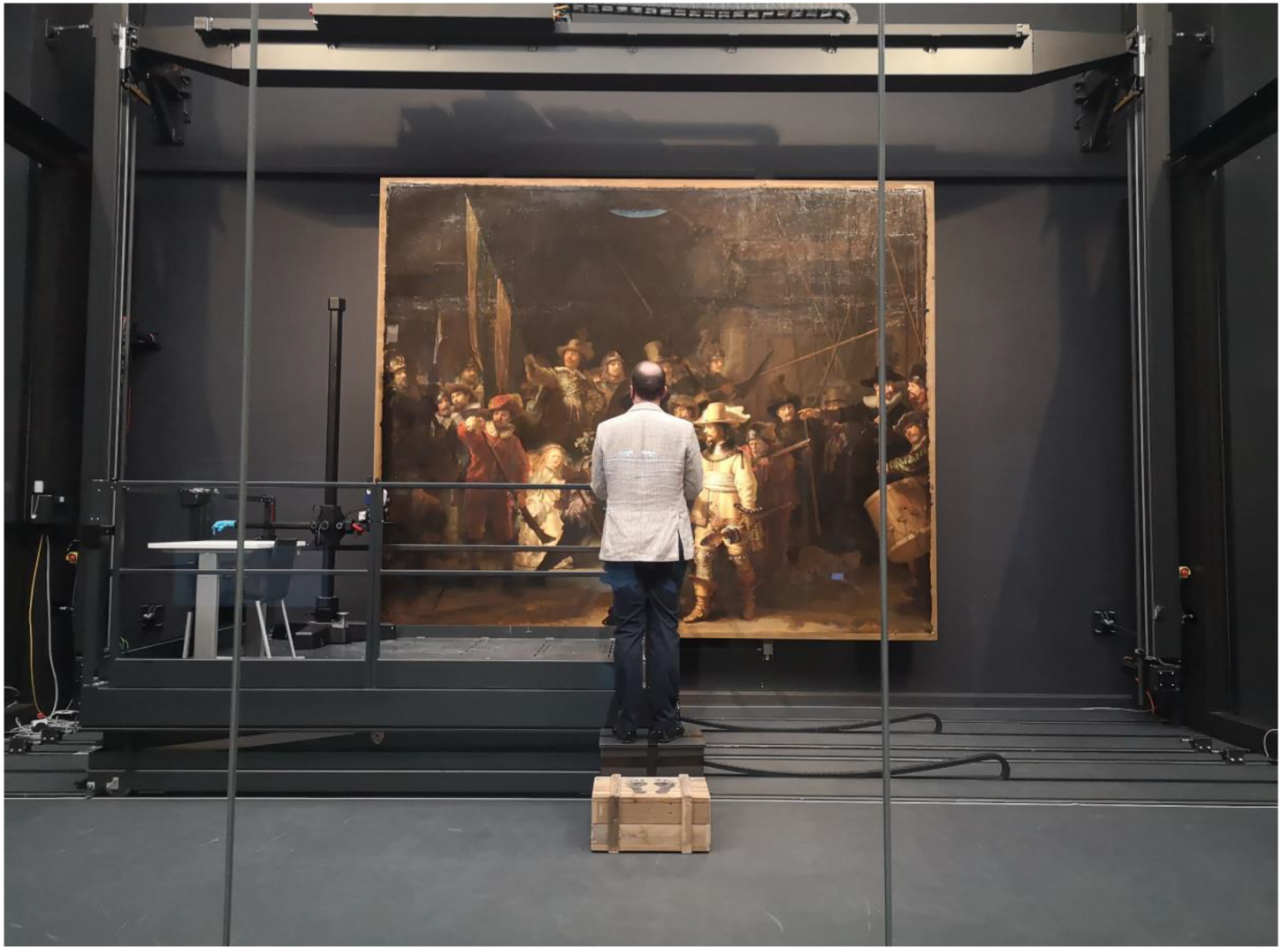
Photo of the experimental setting. The photo was taken from outside the glass chamber in which *The Night Watch* was located.

Participants wore the eye-tracker, which was then calibrated using the one-point Tobii procedure. In the calibration, the participant looked at a calibration card that was held by an assistant. After the calibration, the experimenter verbally repeated the instructions. The experimenter then walked into the adjacent *Gallery of Honour*, and into the glass chamber (Gabrieli et al., 2021) in which *The Night Watch* was located, with the participant following. The participant was asked to step onto a block (L × W × H: 52 × 37 × 40 cm), while still looking down. The front edge of this block was at a distance of 271 cm from *The Night Watch* and positioned against a platform in front of the painting. The experimenter then said *start*, after which the participant started looking at the painting. Figure 2 depicts the experimental setting.

During the experiment, the experimenter was present in the corner of the glass chamber, approximately 3 m behind and 4 m to the right of the participant. The experimenter used a stopwatch to keep track of time since the start of the experiment. The experimenter nudged the participant to keep talking if they were silent (this occurred once in two participants) or talk louder if they spoke softly (this occurred once in three other participants). Five minutes after the start, the experimenter told the participant that the experiment was finished.

The participant then walked back to the initial room and completed a questionnaire that asked about their experience with arts and their expectations before arriving at the Rijksmuseum. At the bottom of the questionnaire, participants were asked not to talk to other participants about what they had experienced. All communication (informed consent, instructions, think-aloud, and questionnaire) was conducted in the Dutch language.

A television crew was present to film the experiment. For most of the participants, the camera operator was outside the glass chamber during the experiment. The camera operator was never in the participant's view during the experiment. For some participants, a security person or camera operator was also present in the other corner of the glass chamber.

### Pre-Processing of Eye-Tracking Data

The raw eye-tracking data were exported to an Excel file using the Tobii Pro Lab software, and the horizontal and vertical gaze coordinates were read into MATLAB R2021b for further processing. The gaze coordinates were available in pixels of the 1920 × 1080 image provided by the eye-tracker camera. Five minutes of data were extracted per participant, starting at the video frame in which the participant's eye-gaze first landed on the painting.

First, data gaps were identified in the recorded eye-movement data. Such data gaps arise, for example, when the participant looked strongly upward, out of the view of the eye-tracker, or due to blinking. For each data gap, the gap was increased by two samples (0.02 s) before the gap and two samples (0.02 s) after the gap to retain high-quality data. Next, the data gaps smaller than 0.5 s were linearly interpolated. Subsequently, a centered moving median filter was applied using a sliding window of 20 samples (0.2 s). Data gaps larger than 0.5 s were not interpolated but left as data gaps because such gaps were regarded as missing rather than the result of a short interruption such as blinking (blinking is known to take 200 ms on average; Caffier et al., 2003).

### Mapping of the Eye-Tracker Gaze Coordinates to a Photo of the Painting

The eye-tracker records the gaze coordinates relative to the participant's head. The participants could freely rotate their heads to obtain different views of the painting. Hence, the gaze coordinates in the eye-tracker image had to be mapped to a frontal image of the painting (Santini et al., 2018). Exploratory analyses indicated that a direct mapping from the eye-tracker image to the frontal image was feasible but not deemed accurate and robust enough.

**Figure 3.**
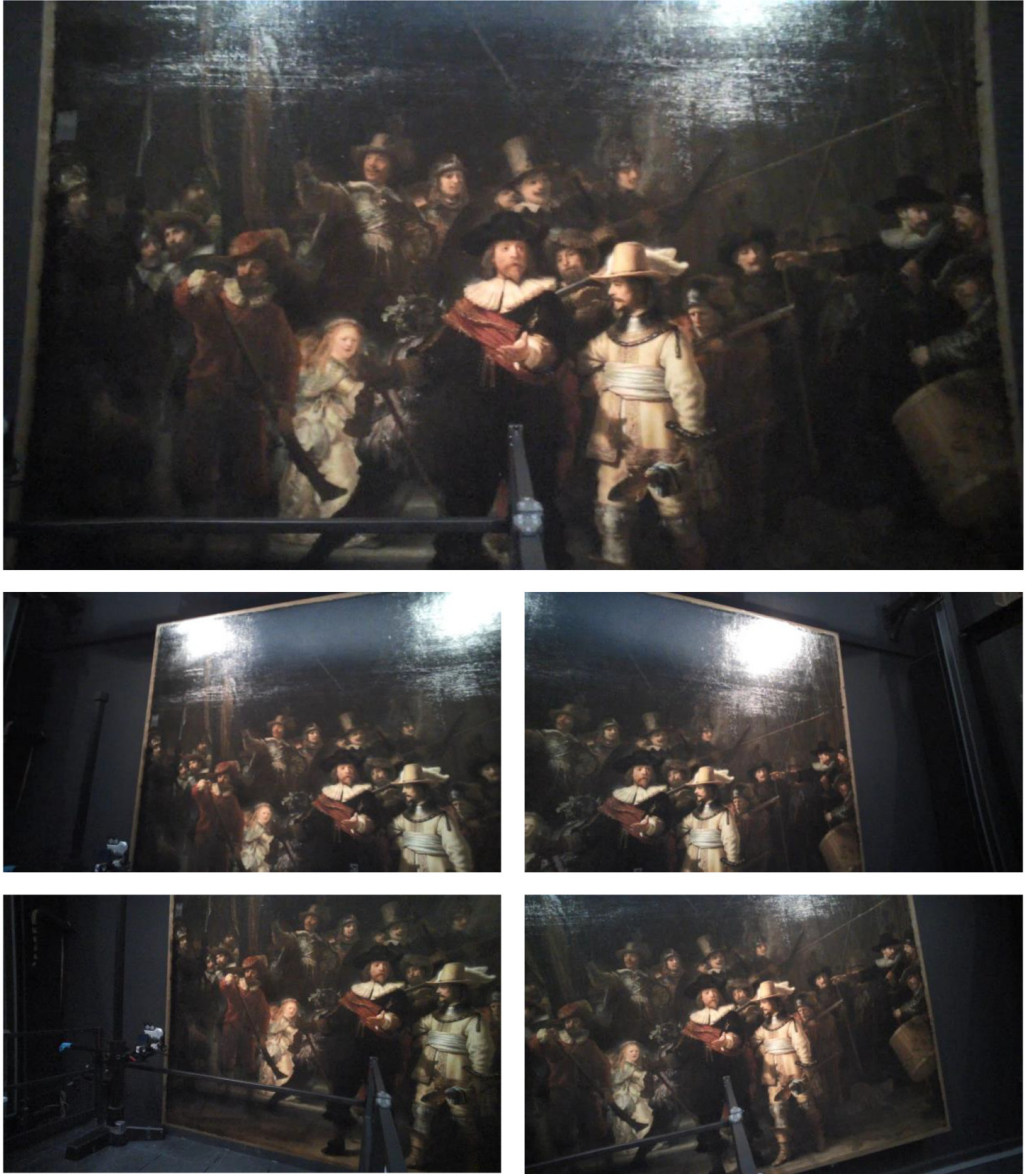
Images recorded with the eye-tracker from the participant's position. The bottom edge of the painting was elevated 60 and 79 cm from the floor during the first and the second day of the experiment, respectively. The participant was standing in front of the rightmost edge of the platform. The lateral distance between the leftmost edge of the painting and the rightmost edge of the platform was 220 and 200 cm during the first and the second day of the experiment, respectively.

A more robust solution was found in a two-stage mapping procedure. In Mapping Stage 1, eye-tracker camera images were mapped to reference images (for a similar template-matching method, see Briechle & Hanebeck, 2001; Onkhar et al., 2021). The reference images were obtained under conditions identical to those in the experiment, thus providing a view of the painting as a participant might have had. A total of nine reference images were used, providing differently angled views of *The Night Watch* (five of them are shown in Figure 3). In Mapping Stage 2, the gaze coordinates in the reference image were mapped to a frontal image of *The Night Watch* (Figure 1, top). The two-stage mapping is explained in more detail below.

### Mapping Stage 1: From Eye-Tracker Camera Image to Eye-Tracker Reference Images

The eye-tracker camera recorded the scene at 25 Hz while eye movements were recorded at 100 Hz. The analysis was conducted for each eye-tracker camera frame. First, the mean gaze coordinate per eye-tracker frame was computed by averaging the four available data points.

**Figure 4.**
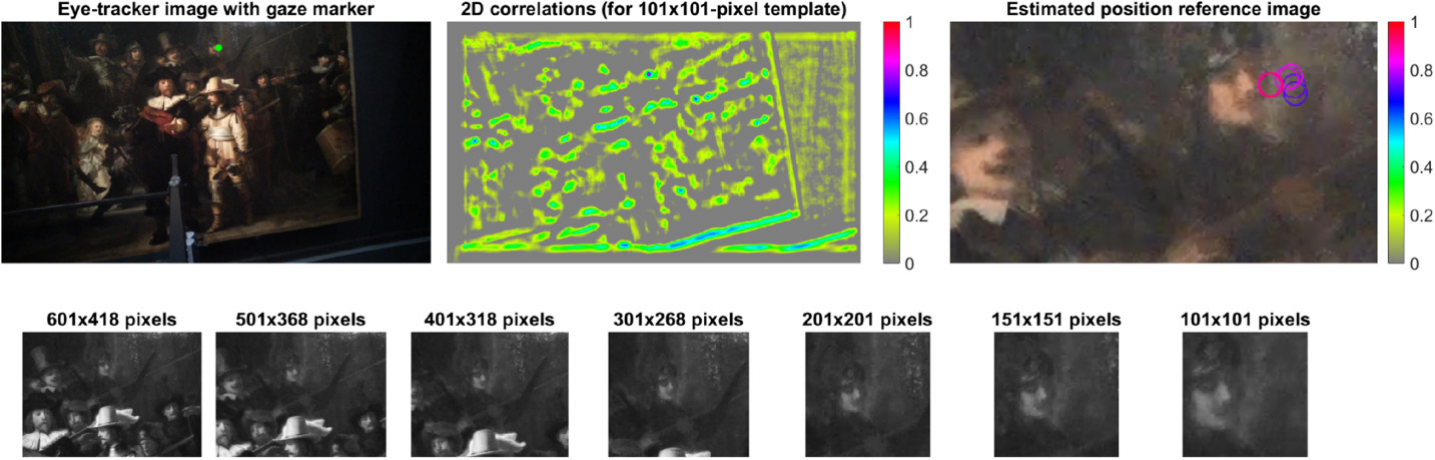
Left top: Eye-tracker camera image (1920 × 1080 pixels) for a random frame with a marker drawn at the measured gaze coordinate. Middle top: Heatmap of correlation coefficients (1920 × 1080 pixels) between one of the templates in grayscale and one of the reference images in grayscale (1920 × 1080 pixels). Right top: Estimated gaze positions, that is, locations of the maximal correlation coefficient, in one of the reference images (here zoomed in to 242 × 136 pixels, for clarity). Seven markers are shown, corresponding to the seven templates. The markers are color-coded according to the maximal correlation coefficient (*r* = 0.72, 0.73, 0.83, 0.82, 0.87, 0.90, 0.93, respectively). Bottom: Templates in grayscale of seven different sizes centered around the gaze coordinate in the eye-tracker camera image. The templates are square-shaped unless they are out of bounds.

Next, templates were cropped around the gaze coordinate, as illustrated in Figure 4 (bottom). The templates had seven different sizes (601 × 601, 501 × 501, 401 × 401, 301 × 301, 201 × 201, 151 × 151, and 101 × 100 pixels), with large templates allowing for a match with the reference images at relatively low precision, and small templates being more challenging to match, but if a valid match is obtained, it usually has high precision. If the participant gazed at a dark region, for example, the template-matching procedure would have to rely on larger templates, as there is not enough variation in the brightness of the small templates.

For each template size, two-dimensional cross-correlations were computed (see Figure 4, middle) using the graphics processing unit (GPU), for accelerated processing. Next, the coordinate of maximal correlation was extracted (see Figure 4, right). Using this procedure, seven candidate coordinates were obtained for each of the nine reference images.

### Mapping Stage 2: From Reference Images to a Frontal Image of The Night Watch

A feature matching method was used to match coordinates of the reference images to a grayscale frontal image of *The Night Watch*. The method used was Oriented FAST and Rotated BRIEF (ORB) (Rublee et al., 2011), which detects corners in the image and is rotation invariant. The scale factor (pyramid decimation ratio) was set to 1.2, while the number of levels was set to 14.

**Figure 5.**
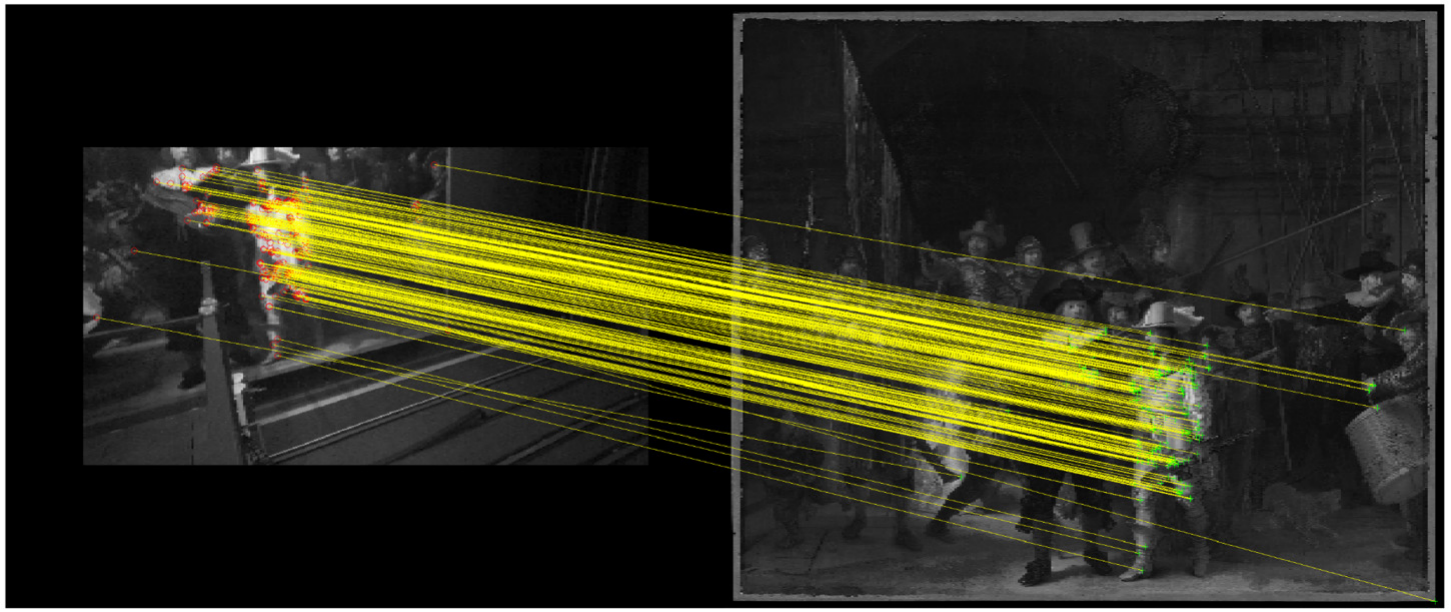
Feature matching results for an eye-tracker reference image (left) and the frontal image of *The Night Watch* (right). Three features were manually added (tip of musket, right eye of the drummer, right corner of the painting).

Figure 5 shows the feature matching results for one of the nine reference images. A total of 245 features were automatically matched. Three feature points were manually added and matched, namely, the bottom right corner of the painting, the tip of the musket of the musketeer (Jan van der Heede), and the eye of the drummer (Jacob Jorisz) (for the names of the people in the painting, see Dudok van Heel, 2009). These features were added to ensure that the edges of the reference image were properly represented. Each of the extra features was repeated 30 times to ensure they had sufficient weight. Thus, a total of 335 feature points were available. The same procedure (but with different manually-added feature points) was used for the other eight reference images.

**Figure 6.**
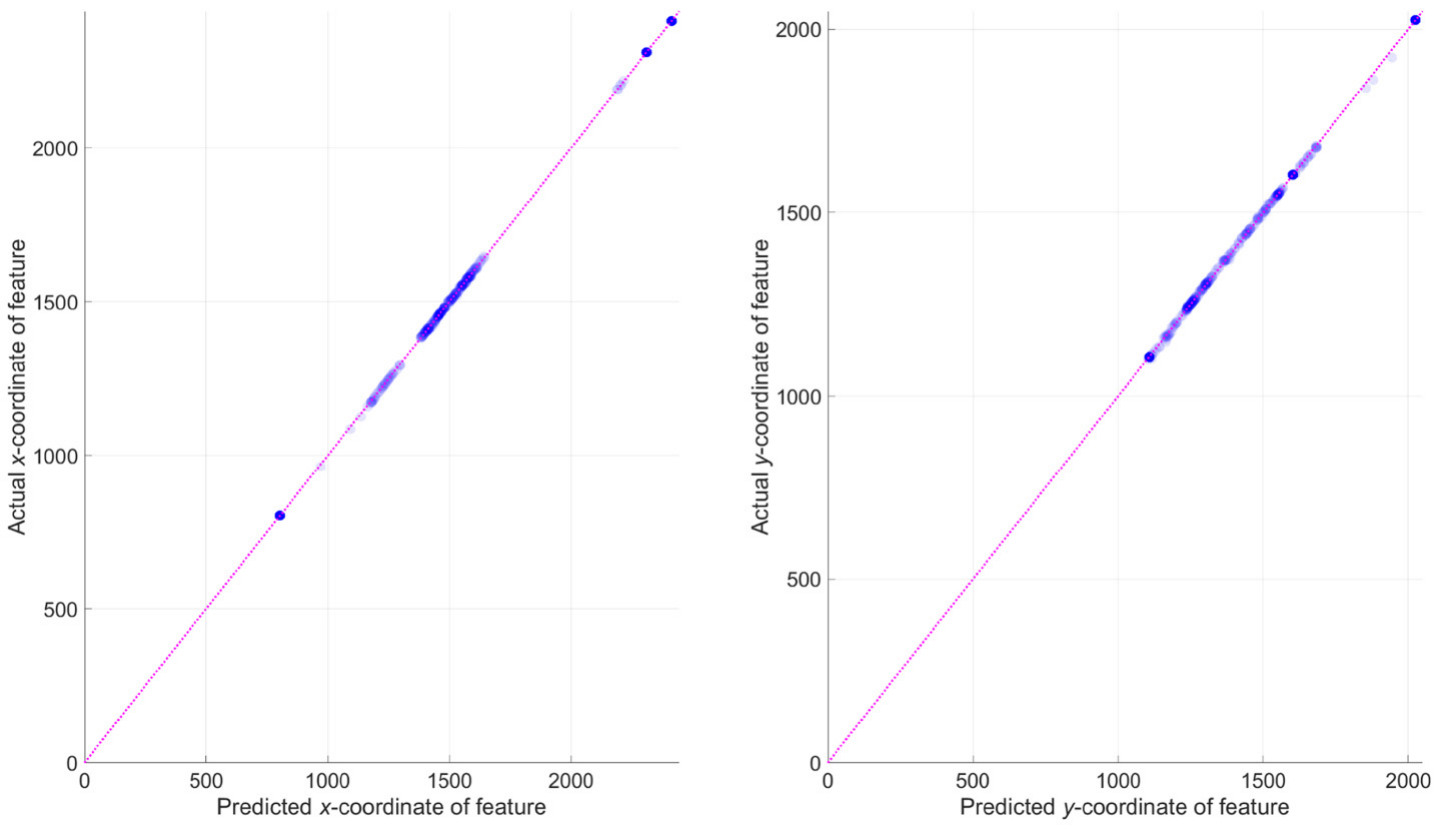
Scatter plot of $x_{actual}$ versus $x_{\;predicted}$ (left) and $y_{actual}$ versus $y_{\;predicted}$ (right). The diagonal line is the line of unity. Markers (*n* = 335) are shown with transparency so that overlap can be seen more clearly. *r *> 0.9999 for the left and right panels.

Next, regression equations were fitted to predict the coordinates in *The Night Watch* image (Figure 5, right) from the coordinates in the reference image (Figure 5, left). More specifically, seven parameter values ( $b_{x1}$… $b_{x7}$) were obtained for equation (1), and seven parameter values ( $b_{y1}$… $b_{y7}$) were obtained for equation (2). Here, $x_{i}$ and $y_{i}$ are the horizontal and vertical coordinates of the *i*th feature. The regression equations provide a linear mapping via $b_{1}$, $b_{2}$, and $b_{3}$, and also include two terms, parameterized by $b_{4}$, $b_{5}$, $b_{6}$, and $b_{7}$, that scale the coordinates to allow stretching. The denominators of the two terms ( $y-b_{7}$ and $x-b_{6}$, respectively) were constrained so that they are lower than −150 pixels or greater than +150 pixels to ensure that the hyperbola did not grow to infinity. The regression equations provide an accurate mapping from the reference image to the frontal image, as illustrated in Figure 6.

(1) $$x_{i,predicted}=b_{x1}+b_{x2}x_{i}+b_{x3}y_{i}+b_{x4}(x_{i}-b_{x6})/(y_{i}-b_{x7})+b_{x5}(y_{i}-b_{x7})/(x_{i}-b_{x6})$$

(2) $$y_{i,predicted}=b_{y1}+b_{y2}x_{i}+b_{y3}y_{i}+b_{y4}(x_{i}-b_{y6})/(y_{i}-b_{y7})+b_{y5}(y_{i}-b_{y7})/(x_{i}-b_{y6})$$

### Determining the Final Coordinate on the Frontal Image of The Night Watch

In Mapping Stage 1, seven coordinates were obtained for each of the nine reference images. These coordinates were mapped to the frontal image of *The Night Watch* using the regression equations described above. This approach yielded a maximum of 63 candidate points in the frontal image. Subsequently, one of these points was selected using a heuristic procedure that ranked the points according to their stability (i.e., do subsequent template sizes for a given reference image yield a similar coordinate estimate?) and their correlation coefficient (i.e., how good is the fit between the template and the reference image?).

The above procedure to determine the final gaze coordinate was repeated for each video frame of each participant. Finally, after processing all frames, a median filter with a window length of 5 (0.2 s) was applied to the final *x* and *y* coordinates to filter out potential outliers.

### Creating the Heatmap

The final heatmap was created by looping through all frames and summing circular patches with a radius of 30 pixels to the frontal image of *The Night Watch* (which measured 2444 × 2048 pixels). The heatmap was also created for different time segments of the experiment (first and last minute of viewing).

**Figure 7.**
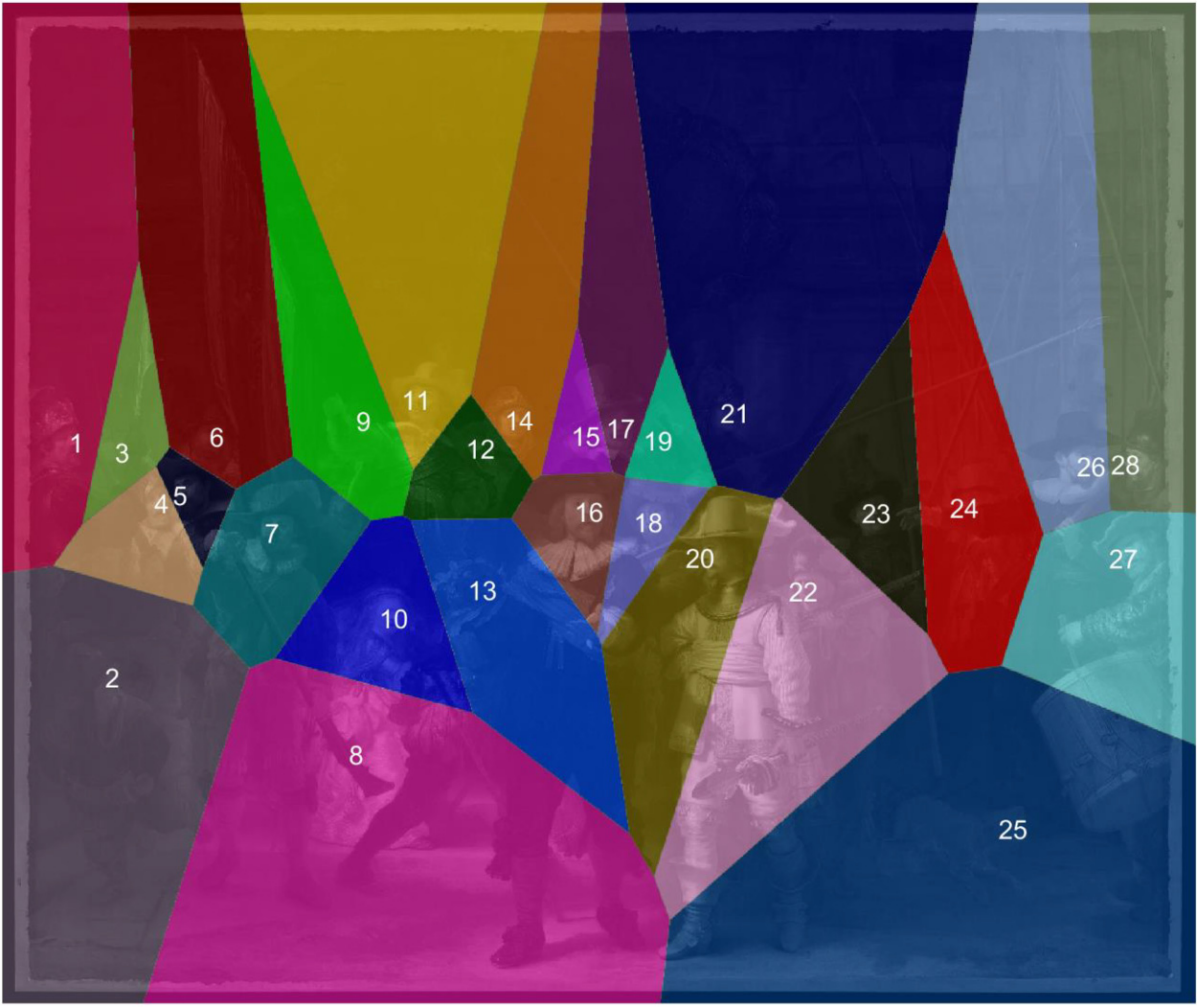
Voronoi cells around all characters’ noses. These cells were used as areas of interest.

The similarity of the heatmaps was judged from the correlation coefficients between the mean heatmap value per pixel for 28 areas of interest (AOIs). The AOIs were created by identifying landmark points for all visible characters (i.e., the noses of all humans, of the dog, and of the chicken on the girl's waist) and creating Voronoi cells around them (see Figure 7). Voronoi cells are more commonly used in eye-tracking research to cluster fixation points (Over et al., 2006). In the current study, Voronoi cells were used to automatically partition the painting into polygonal AOIs. The border of each polygon has an equal distance to two landmark points, and so each Voronoi cell consists of all points closer to the landmark point than to all other landmark points.

Apart from judging the similarity of heatmaps, correlations were calculated between the AOIs’ mean heatmap value, mean local entropy value (as shown in Figure 1), mean grayscale value, and eccentricity (defined as the distance in meters between the AOI's landmark point and the center of the painting). These correlations allowed judging whether texture, brightness, and centrality (Bindemann, 2010; Tatler, 2007) covary with where on the painting people look.

### Experiment Using a Replica of The Night Watch

In order to examine the robustness of our method, we repeated the experiment using the same protocol with 27 new participants in the period between 16 July and 10 September 2021. Participants were recruited from the student population and the teaching and administration staff of the authors’ faculty building at the Delft University of Technology. The recruitment procedure for the replica experiment was somewhat less formal than the recruitment procedure for the real *Night Watch*, with 15 participants receiving the consent form in advance and 12 participants participating directly after recruitment. Again, participants were required to be able to speak Dutch.

The sample consisted of 13 males and 14 females and had a mean age of 34.2 years (range: 25–60 years*, SD* = 10.2, *n* = 25; two participants did not specify their age). Further details about the participants are provided in Table 2. One-third of the participants owned a national Museum Pass, and overall, the participants had a moderate interest in art but had not completed an art-related education. Participants provided written informed consent, and the research was approved by the Human Research Ethics Committee of the TU Delft.

**Table 2.** Responses to the post-experimental questionnaire for the replica *Night Watch* experiment (number of participants and percentages).

**Question**	**Response options**				
	**No**	**Yes**			
Q1. As an adult, I took classes about art.	23	4			
	85%	15%			
Q2. I took part in education about art.	27	0			
	100%	0%			
Q3. I have a Museum Pass.	18	9			
	67%	33%			
	**0**	**1–3**	**4–6**	**7–9**	**10 or more**
Q4. On average, how many times a year do you visit an art exhibition, gallery, or museum (before COVID-19)?	6	9	10	2	0
	22%	33%	37%	7%	0%
	**0**	**1**	**2**	**3**	**4 or more**
Q5. On average, how many hours a week do you create visual art?	21	4	0	1	1
	78%	15%	0%	4%	4%
Q6. On average, how many hours a week do you read about art?	20	6	1	0	0
	74%	22%	4%	0%	0%
Q7. How many times in total have you been to the Rijksmuseum before? (give an estimate)	Mean = 2.0, *SD* = 2.0, Median = 1, *n* = 24				
	**Not familiar at all**	**Somewhat familiar**	**Moderately familiar**	**Very familiar**	**Extremely familiar**
Q8. How familiar are you with history of painting?	9	15	2	1	0
	33%	56%	7%	4%	0%
Q9. How familiar are you with the psychology of eye movements?	14	9	1	3	0
	52%	33%	4%	11%	0%
Q10. How familiar are you with the painting techniques used in *The Night Watch*?	17	6	3	1	0
	63%	22%	11%	4%	0%
	**Strongly disagree**	**Disagree**	**Neither disagree nor agree**	**Agree**	**Strongly agree**
Q11. I have been thinking about *The Night Watch* during the past week.	23	2	0	2	0
	85%	7%	0%	7%	0%
Q12. In the past week, I have looked up information or read about *The Night Watch*.	24	2	0	1	0
	89%	7%	0%	4%	0%

**Figure 8.**
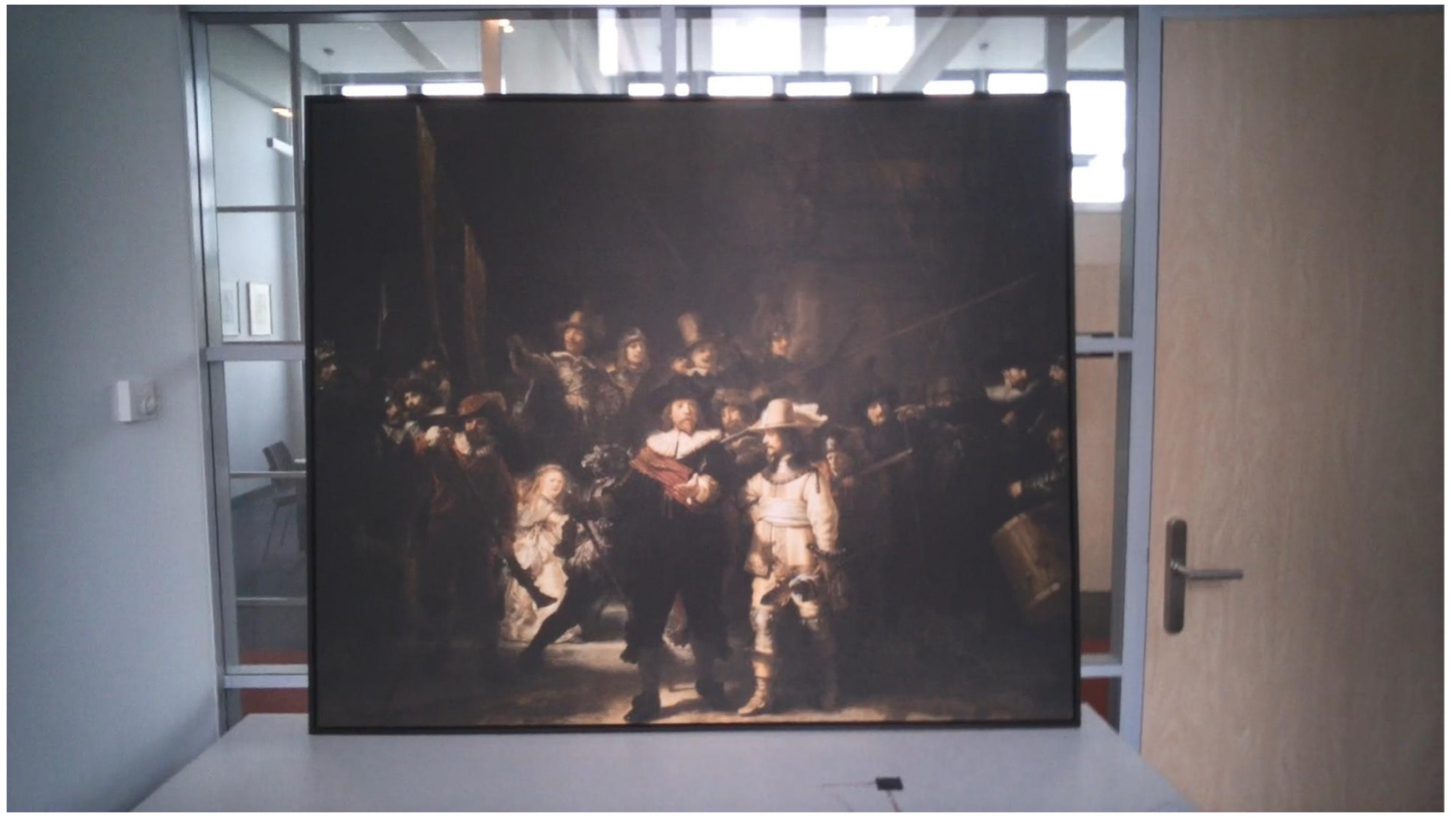
Image recorded with the eye-tracker from the participant's position for the replica study.

Participants looked at a replica of *The Night Watch* of 169.5 × 140.0 cm (measured excluding the frame) that was printed on canvas. They stood at a distance of 175 cm, marked with a circular spot on the ground on which the participants were asked to stand. The experimenter nudged the participant to keep talking if they were silent (this occurred once in two participants) or if the participant thought that the experiment was completed before the 5 min were over (this occurred once in another participant). The heatmap was obtained using the same data processing procedure but with nine different reference images, one of which is shown in Figure 8.

### Processing of Think-Aloud Data

The participants’ verbal utterances were transcribed word-by-word. Incompletely spoken words and the filler words *uh* or *um* were not transcribed. For the experiment with the real *Night Watch*, 12 statements in which the participants referred to the spotlights reflecting on the painting were excluded from the analysis.

Different methods of analyzing the think-aloud data were considered, including thematic analysis, where the researchers extract themes from verbal utterances, without necessarily counting how often those themes were addressed (e.g., Braun & Clarke, 2006). Another option would be to use a fully quantitative method, such as a tabulation or visualization of word frequency and connectedness (e.g., Heikoop et al., 2018). It was reasoned that a thematic analysis would be too imprecise, considering that our study goal was to understand participants’ attentional processes. At the same time, a quantitative content analysis, such as a word cloud, was deemed insufficiently meaningful. Therefore, we opted for a hybrid thematic-content approach, where we defined themes and subsequently counted how frequently participants mentioned target words belonging to those themes.

Seventeen thematic categories of words were created, as shown in Table 3. The themes were based on apparent frequency, identifiability, and semantic distinctiveness. For example, upon transcribing the results, it was noted that participants regularly mentioned the girl in the painting. Corresponding words (e.g., girl, women, daughter) were clearly identifiable as belonging to the same character/theme and semantically distinct from other words (e.g., dog, people in general).

**Table 3.** Seventeen themes and their corresponding words (translated from Dutch to English).

Theme	Words
Admiration	beautiful, cool, enjoy, fantastic, fascinating, great, impressive, nice, special, splendid, unique, wonderful, wow
Central figures	ally, Banninck Cocq, boss, central, foreground, front, general, henchman, important, in front, king, kings, leader, lieutenant, main character, main figure, man on the right, merchant, merchants, Ruyter, middle, most important, nobility, noblemen, protagonists, speaker
Chicken	bird, chicken, pigeon, rooster, trophy
Clothing	ankle boots, army helmets, belt, bird feather, boots, buttons, cape, clothes, clothing, collar, collars, costume, decorative edges, drawstring bag, dress, edges, embellishment, embellishments, embroidery, fabric, fabrics, feather, feathers, folds, glove, gold stitching, hand, hat, headgear, heels, helmet, helmets, jacket, jackets, kaftan, lace, laurel wreath, neck-sash, outfit, pleats, plume, plumes, pretty things, puffers, robe, robes, sash, scarf, scarf-like, scarves, shoes, sleeve, sol, suit, suits, uniform, vest, wreath neck
Color	blue, bluish, brown, brownish, color, colorful, colors, gold, golden, gray, green, greenish, light yellow, red, yellow
Contrast	contrasting, contrasts, highlighted, lighter, white
Darkness	black, dark, darkness, dimmer, gloomy, shading, shadow, shadow effects, shadows, shadowy
Detail	brush, brushstrokes, detail, detailed, detail-rich, details, level of detail, photo, realistic, sharp, sharper, texture
Dog	cat, dog
Drum	drum, drummer, drumming, drums, instruments, musical instrument, musician
Faces	beard, bearded, expression, expressions, eye, eyes, face, faces, facial expressions, goatee, head, heads, mustache
Girl	angel, angelic, daughter, girl, girls, lady, maid, woman
Lighting	expose, flash, highlighted, illuminated, light, light beam, light effects, light source, lighting, shines
Mission	attack, battle, besieged, combat, conquer, defend, enemy, fight, fighting stance, guard, hunt, military, militia, mission, protect, pull out, revolt, revolution, shot, stir up, the watch, victory, war, win, won
Night Watch	Night Watch
People	character, characters, figures, males, man, men, mister, night watchmen, people, persons, soldiers, watchman, watchmen
Weapons	armor, arrow, arrows, banner, beams, flag, flags, gun, guns, harpoon, lance, lances, musket, patch, pawls, pistol, spear, spearheads, spears, spear-thing, stick, sticks, sword, sword-like, swords, weapon, weapons

For each theme, we extracted the number of words spoken per participant and the mean elapsed time of the spoken words. The number of words spoken per participant was compared between the real and replica painting using Welch's *t*-test. A Bonferroni correction was applied, meaning that the alpha value was set to 0.05/17≈0.003.

## Results

### Real Night Watch

For one of the 21 participants, 202 instead of 300 s of data was available due to an empty battery of the eye-tracker. Eye-tracking data of participants were available for 95.1% of the time on average (*SD* = 4.4%, min = 88.1%, max = 99.8%). The missing data consisted of the aforementioned gaps of 0.5 s or longer, for example, due to blinks.

**Figure 9.**
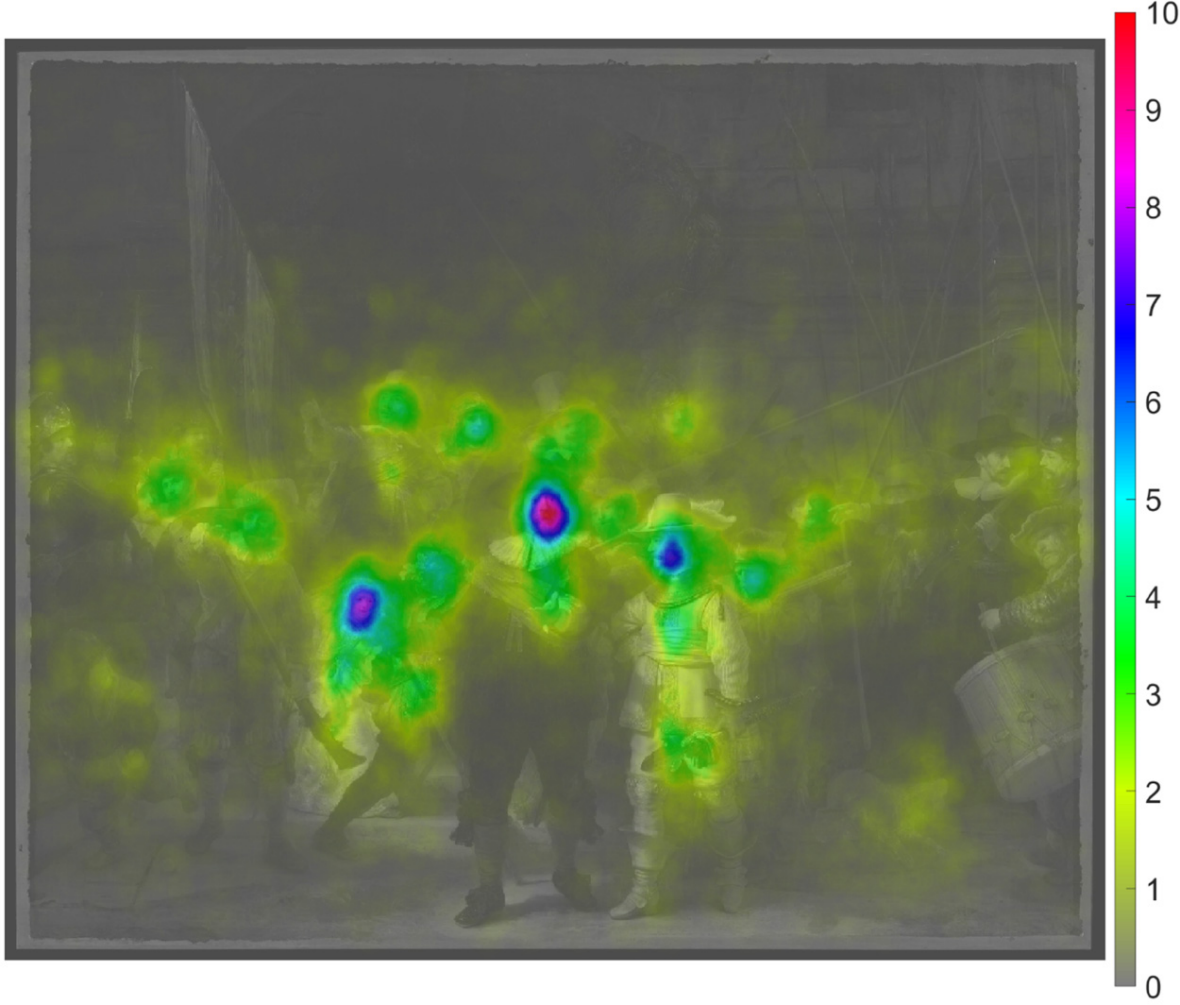
Heatmap of the 21 participants combined for the real *Night Watch*. The heatmap was normalized by dividing by the number of patches and multiplying by 1000.

The heatmap of all participants is shown in Figure 9. It can be seen that attention was not uniformly distributed, but concentrated on specific elements. First, the faces of the people in the painting attract attention. The faces of the leading men, captain Frans Banninck Cocq and lieutenant Willem van Ruytenburch, received the most attention. It is also striking that participants often focused on the girl's face (the mystical and bright appearing figure). Furthermore, the lieutenant's attire and lance (partisan) attracted much attention, as did the girl's attire. Dark clothing attracted little attention; it is noteworthy that the suits of captain Frans Banninck Cocq, sergeant Reijnier Engelen, and musketeer Jan van der Heede, were virtually ignored.

**Figure 10.**
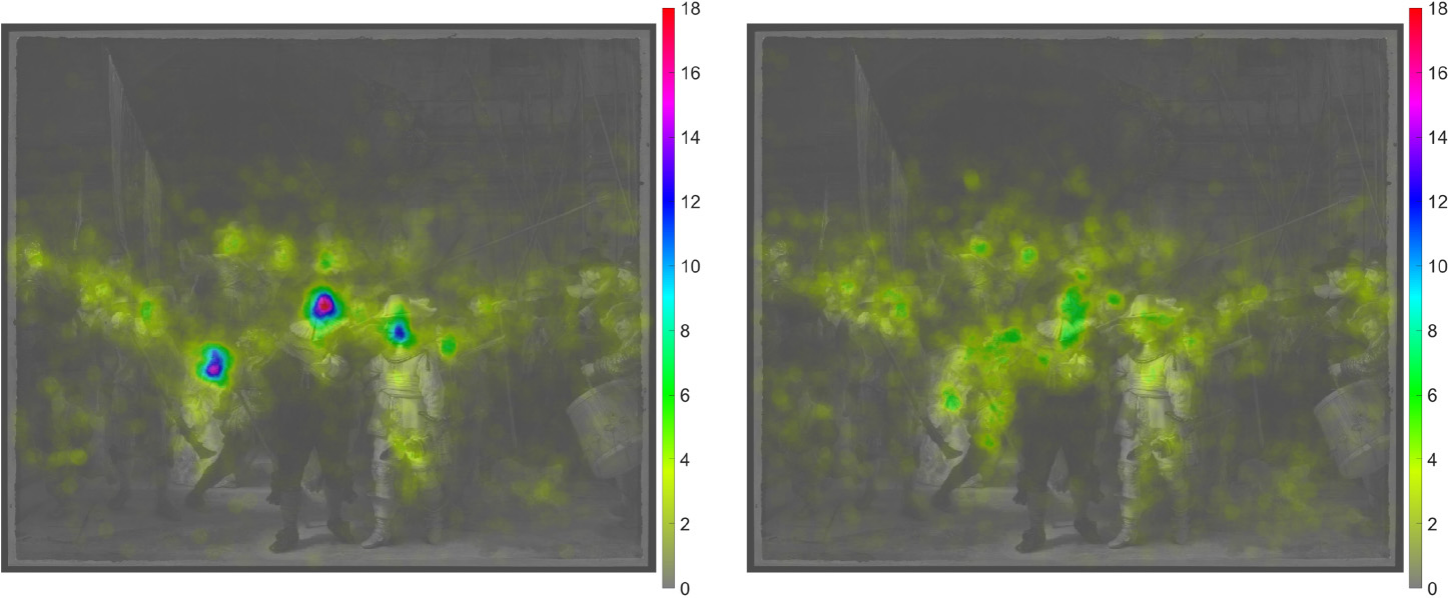
Left: Heatmap for the first 60 seconds of viewing of the real *Night Watch*. Right: Heatmap for the last 60 seconds of viewing of the real *Night Watch*. The heatmaps were normalized by dividing by the number of patches and multiplying by 1000. Note that the color bar ranges from 0 to 18 but from 0 to 10 in the previous heatmap.

It was examined whether participants’ viewing behaviors changed with time on task. Figure 10 shows the heatmap for the first versus last minute of the experiment. It can be seen that, in the beginning, participants focused on the key figures, while, later in the experiment, other elements of the painting, such as the dog, the drummer, and the girl's attire, received more attention.

**Figure 11.**
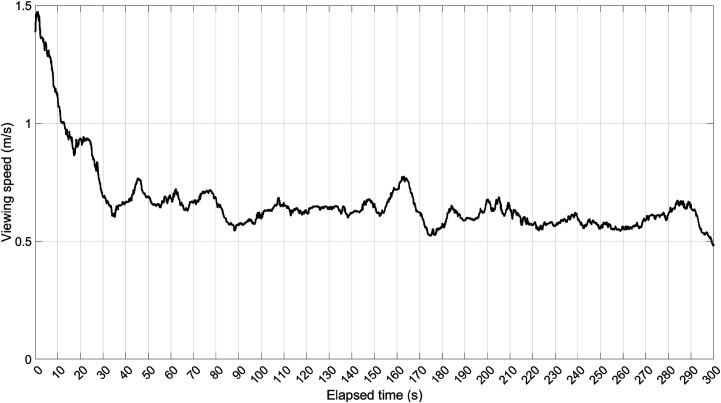
Viewing speed of the real *Night Watch*, that is, the speed of movement of the gaze point on the surface of the painting. This figure represents the median of all participants, which was subsequently filtered using a median filter with a window length of 250 (10 s).

An analysis of the viewing speed across the painting shows that the participants’ eyes were more active in the first minute compared to the later part of the experiment (Figure 11). A likely explanation is that, in the beginning, participants made large saccades (i.e., high-velocity eye movements) to orient themselves on different portions of the painting, after which their eyes remained relatively stable in an attempt to analyze details.

**Figure 12.**
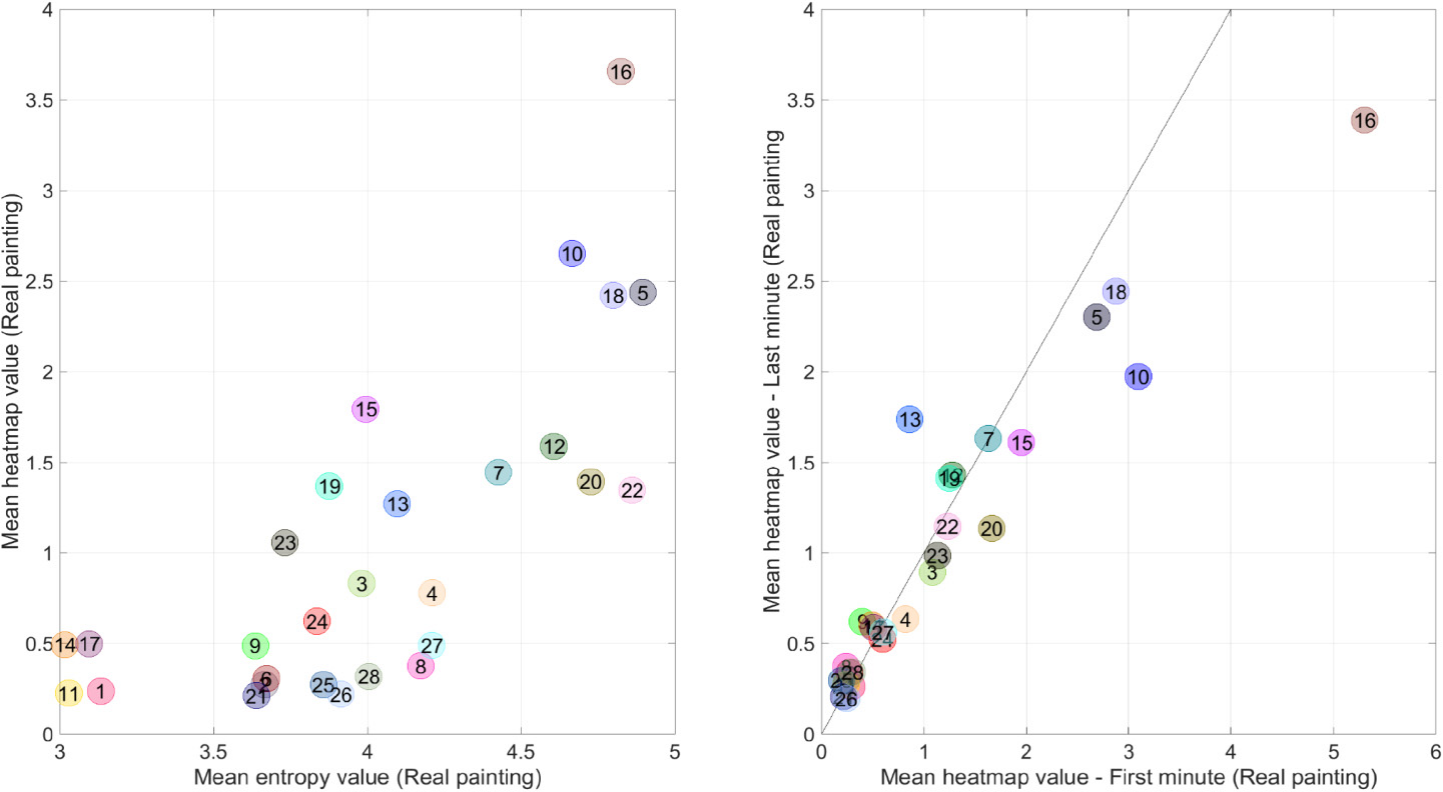
Left: Scatter plot of the mean heatmap value (real painting) versus the mean local entropy value (real painting) per area of interest. Right: Scatter plot of the mean heatmap value (real painting) for the last versus first minute of viewing. The diagonal line is the line of unity. The numbers correspond to the areas of interest (AOIs) shown in Figure 7. It can be seen, for example, that AOI 16 (the face of captain Frans Banninck Cocq) received less attention in the last minute compared to the first minute.

Table 4 shows a correlation matrix of the participants’ attention per pixel per AOI. We found support for the hypothesis that texture (entropy) and brightness attracted the viewers’ attention, with correlation coefficients of 0.74 and 0.64/0.81, respectively (see Figure 12, left, for a scatter plot corresponding to the former correlation). Additionally, it was found that eccentricity was negatively correlated (*r* = −0.53) with the heatmap values. In other words, the central AOIs received more attention than the AOIs that were located near the edges of the painting. This association is illustrated in Figure S1 of the Supplemental material.

**Table 4.** Pearson correlation matrix among mean heatmap values, mean local entropy, mean grayscale value, and eccentricity per area of interest (*n* = 28).

	1	2	3	4	5	6	7	8	9
1. Mean heatmap value (Real painting)									
2. Mean heatmap value, first minute (Real painting)	0.98								
3. Mean heatmap value, last minute (Real painting)	0.98	0.95							
4. Mean heatmap value (Replica)	0.95	0.91	0.94						
5. Mean heatmap value, first minute (Replica)	0.96	0.99	0.95	0.91					
6. Mean heatmap value, last minute (Replica)	0.82	0.75	0.82	0.95	0.73				
7. Mean entropy value (Real painting)	0.74	0.68	0.70	0.70	0.67	0.63			
8. Mean grayscale value (Real painting)	0.64	0.64	0.56	0.56	0.62	0.45	0.84		
9. Mean grayscale value (Replica)	0.81	0.76	0.78	0.71	0.75	0.60	0.61	0.55	
10. Eccentricity (Real painting)	−0.53	−0.48	−0.55	−0.45	−0.49	−0.39	−0.14	−0.05	−0.82

*Note.* The mean entropy value (real painting) and mean grayscale value (real painting) were computed for the image shown in Figure 1. The mean grayscale value (replica) was computed for the image shown in Figure S2 (see Supplemental material) after setting the border, which depicted a bright background, to black.

### Replica of *The Night Watch*

Eye-tracking data of participants were available for 96.1% of the time on average (*SD* = 3.2%, min = 89.9%, max = 99.7%), which are numbers similar to those obtained for the real painting.

**Figure 13.**
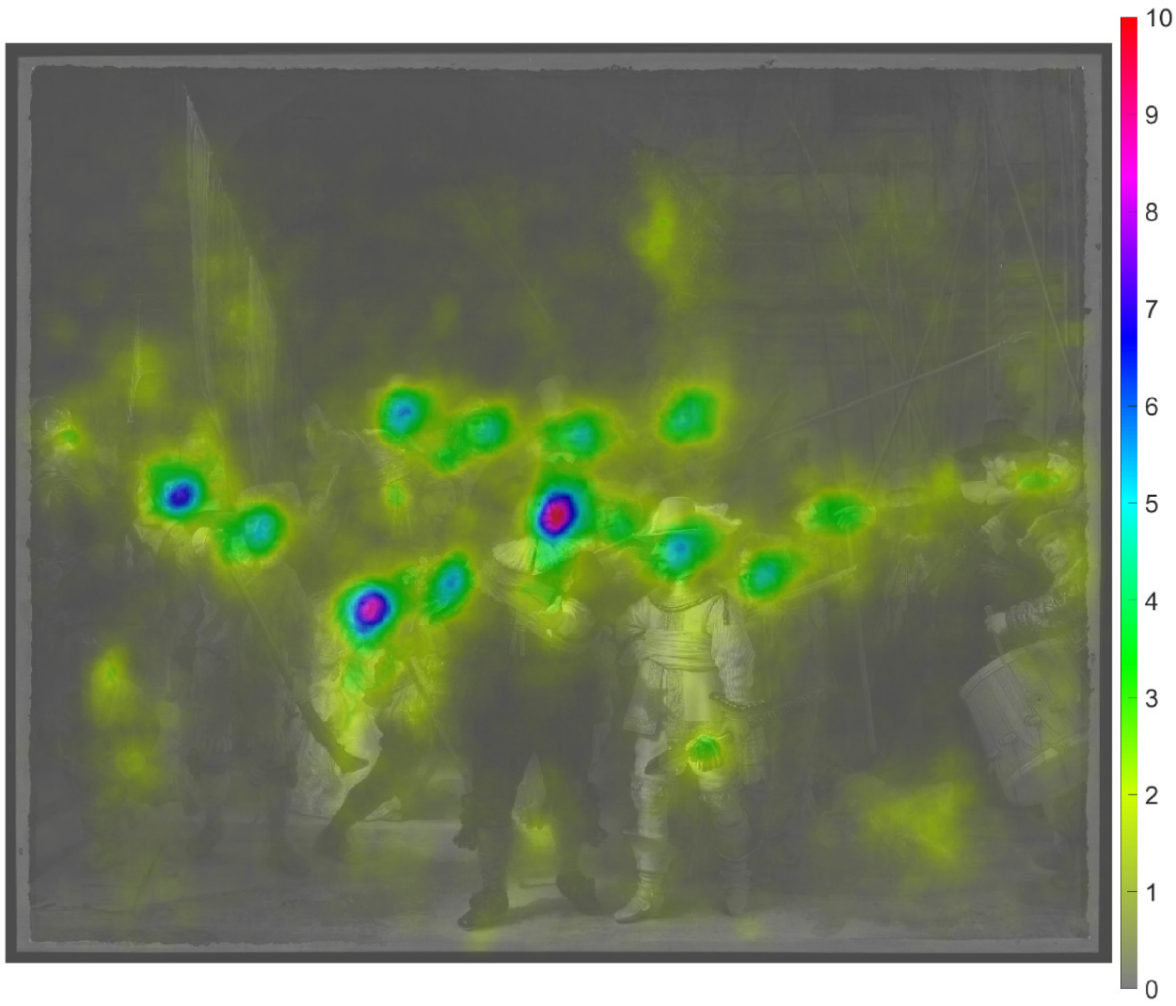
Heatmap of the 27 participants combined for a replica of *The Night Watch*. The heatmap was normalized by dividing by the number of patches and multiplying by 1000.

**Figure 14.**
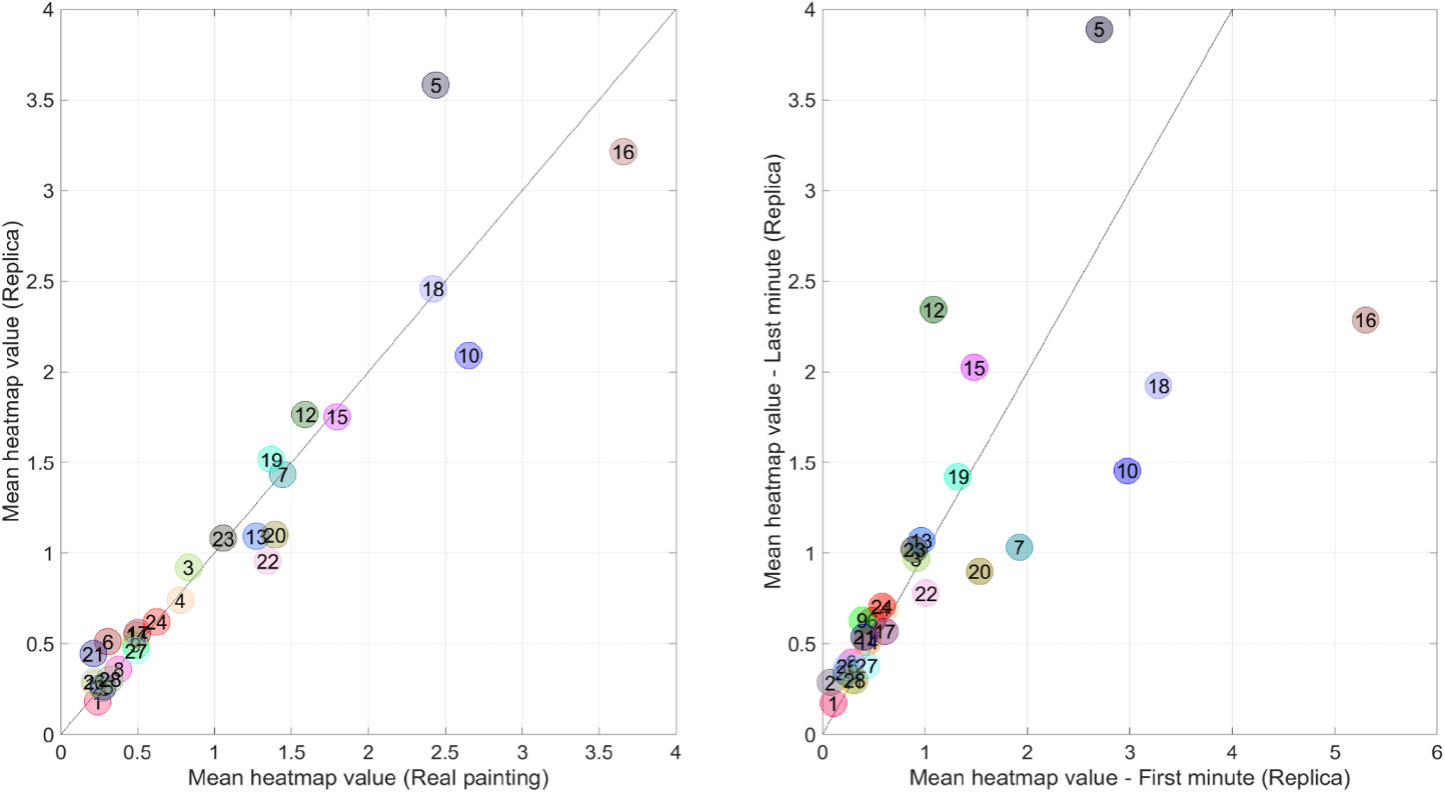
Left: Scatter plot of the mean heatmap value for the replica versus the real painting. Right: Scatter plot of the mean heatmap value for the last versus first minute of viewing for the replica painting. The diagonal line is the line of unity. The numbers correspond to the areas of interest shown in Figure 7.

The heatmap of the experiment for the replica study is shown in Figure 13. There is a strong similarity to the heatmap of the real *Night Watch* (Figure 9) (see Table 4, *r* = 0.95). However, different faces received different amounts of attention between the real and replica painting. In particular, the face of Harman Jacobsen Wormskerck (AOI 5) received more attention, while the main character Willem van Ruytenburch (AOI 20) received less attention compared to the real painting (see Figure 14, left, for a scatter plot). Figure 14 (right) also illustrates that the main figures, such as the captain (AOI 16) and the girl (AOI 10), received more attention at the beginning than at the end, which corresponds to the findings for the real painting (Figure 10 and Figure 12, right).

It is noteworthy that the mean grayscale levels of the AOIs of the replica correlated strongly with the heatmap values of the real painting (*r* = 0.81) and with eccentricity (*r* = −0.82; see Figure S1), while grayscale levels of the replica and real painting correlated only moderately (*r* = 0.55). A presumed explanation is that the grayscale differences in the replica are more realistic than the grayscale differences in the photo of the real painting shown in Figure 1, which appears brightly illuminated and of high contrast.

### Think-Aloud Results for Real Painting Versus Replica

Participants uttered a similar number of words in front of the real painting (*M* = 388, *SD* = 169; *n* = 21) as in front of the replica (*M* = 458, *SD* = 181, *n* = 27), *t*(44.4) = 1.36, *p* = 0.180. Participants also spoke a similar number of target words (i.e., sum of counts of words of the 17 themes) for the real painting (*M* = 43.4, *SD* = 23.8; *n* = 21) as for the replica (*M* = 41.3, *SD* = 19.5, *n* = 27), *t*(38.4) = 0.33, *p* = 0.741.

**Figure 15.**
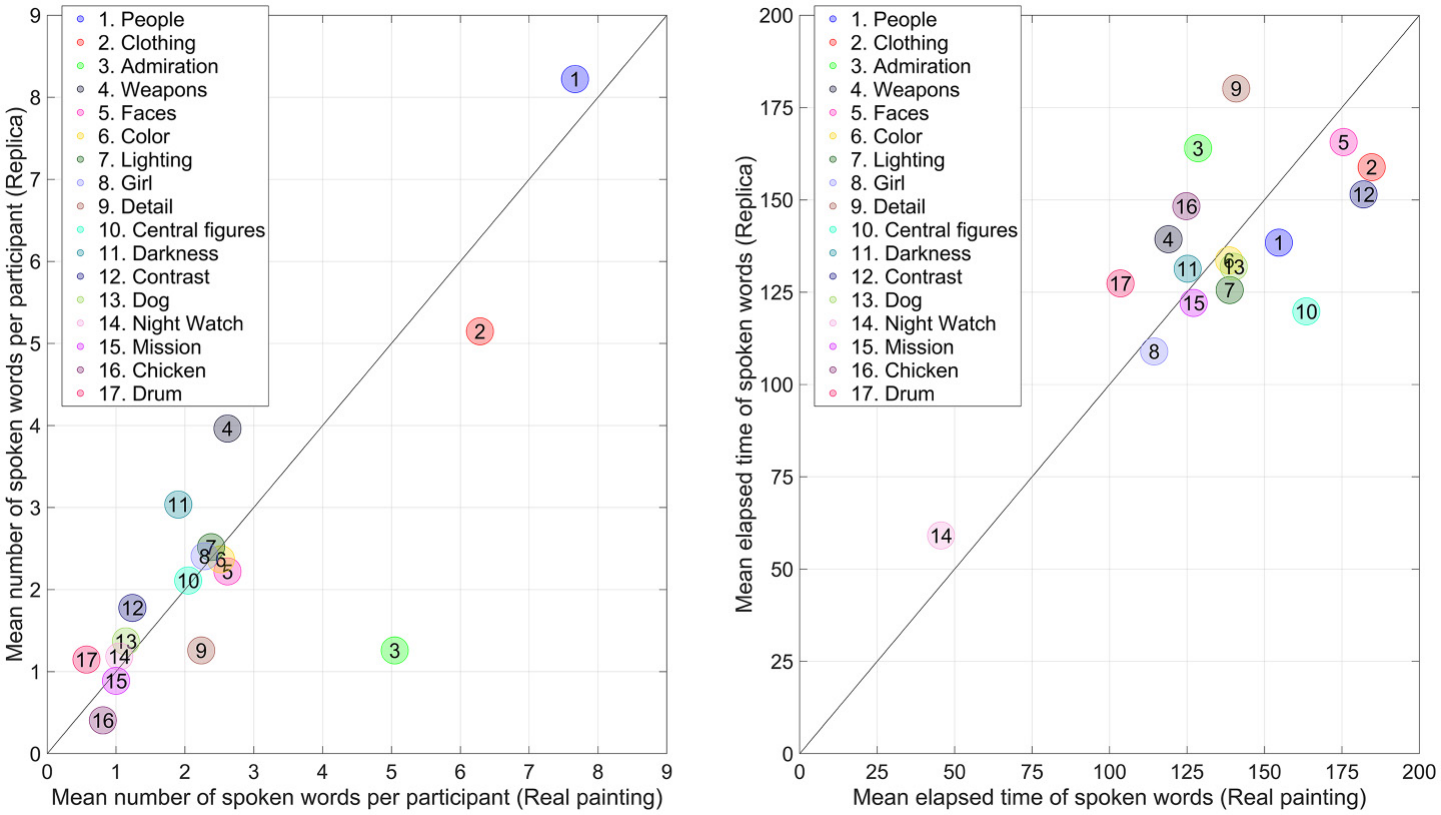
Left: Scatter plot of the mean number of spoken words per participant per theme for the experiment with the replica *Night Watch* versus the real *Night Watch*. The numbers of the themes are sorted according to the mean number of spoken words in the real painting in descending order. Right: Mean elapsed time of spoken words for the replica *Night Watch* versus the real *Night Watch*. The diagonal line is the line of unity.

Regarding the think-aloud data, the mean number of spoken words of the 17 themes showed a high congruence between the replica study and the experiment on the real *Night Watch* (*r* = 0.83, *n* = 17) (see Figure 15, left). However, there was a large difference in regard to the category Admiration, where the number of spoken words per participant was 5.05 (*SD* = 4.10, *n* = 21) in the real painting but only 1.26 (*SD* = 1.85, *n* = 27) in the replica, *t*(26.3) = 3.93, *p *< 0.001.

With regard to the elapsed time of the spoken words, participants appeared to express recognition of the painting *The Night Watch* early in the experiment. Prominent features such as the girl and the drummer were also recognized early. Later in the experiment, participants' utterances focused on faces and clothes (see Figure 15, right).

## Discussion

The purpose of this research was to explore how people look at *The Night Watch*. In this work, we introduced a method to map eye movements, as recorded with a mobile eye-tracker, to a frontal image of the painting. A two-stage mapping procedure was employed, in which the eye-tracker camera image was first mapped to reference images, which were then mapped to the frontal image.

The heatmaps and corresponding analyses of AOIs showed that bright and textured elements in *The Night Watch* attract the observer's eyes. These findings are in line with modern research into scene exploration and painting viewing, which has shown that salient features (Le Meur et al., 2020; Quian Quiroga & Pedreira, 2011; Xu et al., 2010) including faces (Bindemann et al., 2005; Massaro et al., 2012; Theeuwes & Van der Stigchel, 2006) attract attention. What is worth noting is that Rembrandt mastered these techniques and managed to bring out the prominent figures (captain and lieutenant, and the girl, the company mascot) long before the onset of eye-tracking research or experimental psychology as a discipline.

A causal relationship between texture, brightness, and centrality, on the one hand, and attention distribution, on the other, cannot be established conclusively. The current study showed that texture (i.e., entropy value), brightness (grayscale value), and centrality (i.e., eccentricity) were strongly correlated with each other and with the observers’ attention distribution, with eccentricity being the weakest of the three predictors. It is possible that centrality is not of key importance, considering that some of the characters in the painting, such as the girl, attracted attention even though not positioned in the middle. Apart from this, it is possible that factors not quantified so far, such as the interplay of lines of the different weapons (for a discussion, see Wijnbeek, 1944, pp. 64–71) contributed to the viewer's attention being directed to the two main characters. In that sense, our study of a real painting should be seen as complementary to research on computer screens, where paintings can be manipulated digitally and hypotheses can be examined in a more controlled manner (DiPaola et al., 2013).

In our study, we used a concurrent think-aloud method, which may have caused altered viewing behaviors relative to viewing the painting without a secondary task. Experimental research on this topic suggests that thinking aloud increases the likelihood that task-relevant screens are glanced at (Jo & Stautmeister, 2011; Ogolla, 2011; Prokop et al., 2020). In the same vein, it may be expected that in normal viewing without think-aloud task, observers will be more inclined to engage in mind wandering and temporarily look away from the painting. It is also acknowledged that the definition of our themes, as depicted in Table 3, is somewhat subjective and may not correspond to how others would construct them. For example, a reviewer suggested merging some of the themes or adding themes, such as a theme that captures the historical context. An analysis of the transcripts, which are available in the Supplemental material, showed that some participants did indeed use words such as “past,” albeit infrequently. Relatedly, the frequency of keyword usage does not capture the phenomenological experience of the participants. In particular, a much-discussed topic is that Rembrandt, through his use of shadow, composition, and choreography, managed to bring out a certain phenomenological experience, previously referred to as “sprong” (see also Van de Wetering, 2011, pp. 62–63 and Taylor, 2013; based on Van Hoogstraeten, 1678). From the results of the think-aloud, it was noticeable that some participants experienced the dynamics of the scene, for example, “*and it seems like they’re all telling a secret one way or another*” and “*many people actually point at things or are talking to someone as if they want to show something.*” The determinants of this “sprong” would deserve further investigation.

The think-aloud method revealed that the participants admired the real *Night Watch* more than the replica. This effect may have been caused by the painting itself but also by the context of the museum (Brieber et al., 2014; Krukar & Dalton, 2020; Specker et al., 2017; see Pelowski et al., 2017, for a review of the factors that determine people's experience of art in a museum). Although the participants’ level of admiration was substantially different between the real painting and the replica painting, the heatmaps were very similar (*r* = 0.95, or *r* = 0.99 when considering only the first minute of viewing). The eye movement analysis and think-aloud protocol showed that participants first focused on the (faces of the) key figures and rapidly scanned the painting, followed by a more concentrated viewing of other faces and clothing. These findings are in line with more general literature showing that observers first tend to make large eye movements, followed by finer ones (Over et al., 2007; Unema et al., 2005). The differences between the heatmap of the real and replica *Night Watch* may be explainable by the quality of the canvas print of the replica, where the face of Wormskerck (AOI 5) in the replica appears to stand out compared to the other characters in his vicinity (see Figure S2 in the Supplemental material). Another reason may be that it is less effortful to scan the periphery of the smaller painting since no large head movements are required. Previous research suggests that the physical effort required affects the likelihood of a certain area being glanced at (Eisma et al., 2018; Wickens, 2008).

Our mapping method is deemed robust and accurate. Robustness was illustrated through the replica experiment on the smaller version of the painting, where the mapping method worked reliably without making any adjustments to the algorithm or parameters. The accuracy of the mapping was demonstrated by the template matching (see Figure 4 demonstrating an accurate mapping of a gaze point) and the translation at an acute angle to a frontal image of the same painting (see Figure S2 in the Supplemental material). Although the mapping method appears to be accurate, with only small discrepancies between the gaze points in the eye-tracker image and frontal image, the eye-tracker itself may be a larger limiting factor, with an accuracy of a few degrees in dynamic conditions (Onkhar et al., 2021). In addition, for the real *Night Watch* study, data appeared to be lost when the participant rotated the eyes strongly upward (e.g., to look at the upper part of the painting without co-rotating the head) because the eye rotation then fell outside the range of the eye-tracker. It is further noted that the template matching procedure was computationally intensive, taking about 30 min to process 1 min of video on a PC with a powerful graphics card. However, the cross-correlation method, which absorbs most of the computation time, could function faster by making it hone in on candidate regions instead of computing the correlation coefficient for every pixel (e.g., Mahmood & Khan, 2012).

A limitation of our study was that participants were standing against a platform, and some of its metal structure blocked the view of the painting (i.e., between the legs of captain Banninck Cocq and the left bottom part, see Figure 3). The platform was part of a research project and could not be removed. Also, participants stood relatively close to the painting, 2.7 m away, while the spotlights appeared to be tuned for optimal viewing from a larger distance (cf. Figure 2). Light from ceiling spotlights reflected on the upper part of the painting, which may have prevented participants from looking at the upper part of the painting. On the other hand, it can be argued that *The Night Watch* in the Rijksmuseum in the past, as well as before in the Musketeers’ Meeting Hall (Kloveniersdoelen) was also seen up close (see De Bruyn Kops et al., 1976 and Martin, 1947, for photos and drawings). At the same time, it can be argued that these limitations are of little consequence; the heatmaps of the real painting and that of the replica were very similar, suggesting that salience was more important than the conditions in which the experiment was performed.

There are other differences to be noted in comparison to Rembrandt's time, such as the fact that the painting was reduced in size in or just after 1715 to fit it between two doors (Haverkamp-Begemann, 1982), an issue that can affect the meaning of our eccentricity measure. Also, the painting has undergone various restorations and has seen different layers of varnish (Van Duijn & Kok, 2016), and it normally has an impressive frame (De Bruyn Kops et al., 1976), which was absent at the time of our experiment. In any case, we expect that the method we have introduced here can be useful in future research on paintings and other art objects. Research on viewing behavior with real paintings, especially large paintings, is rare, and more such research is recommended. Future research is also encouraged in more unconstrained settings to examine how participants distribute their attention between the painting and other features such as labels (Reitstätter et al., 2020, 2022) and the examination of other types of gaze patterns such as changes of perspective (Eghbal-Azar & Widlok, 2013).

## Supplemental Material

sj-docx-1-pec-10.1177_03010066221122697 - Supplemental material for How do people distribute their attention while observing *The Night Watch*? Click here for additional data file. Supplemental material, sj-docx-1-pec-10.1177_03010066221122697 for How do people distribute their attention while observing *The Night Watch*? by Joost C. F. de Winter, Dimitra Dodou, and Wilbert Tabone in Perception
