# Supplementary material for: How do people distribute their attention while observing The Night Watch?
Source: Perception. 2022 Sep 29;51(11):763–88. doi: 10.1177/03010066221122697 (PMC9557837; doi:10.1177/03010066221122697)
Supplement: sj-docx-1-pec-10.1177_03010066221122697 - Supplemental material for How do people distribute their attention while observing The Night Watch? [file sj-docx-1-pec-10.1177_03010066221122697.docx]

**Supplementary Material**


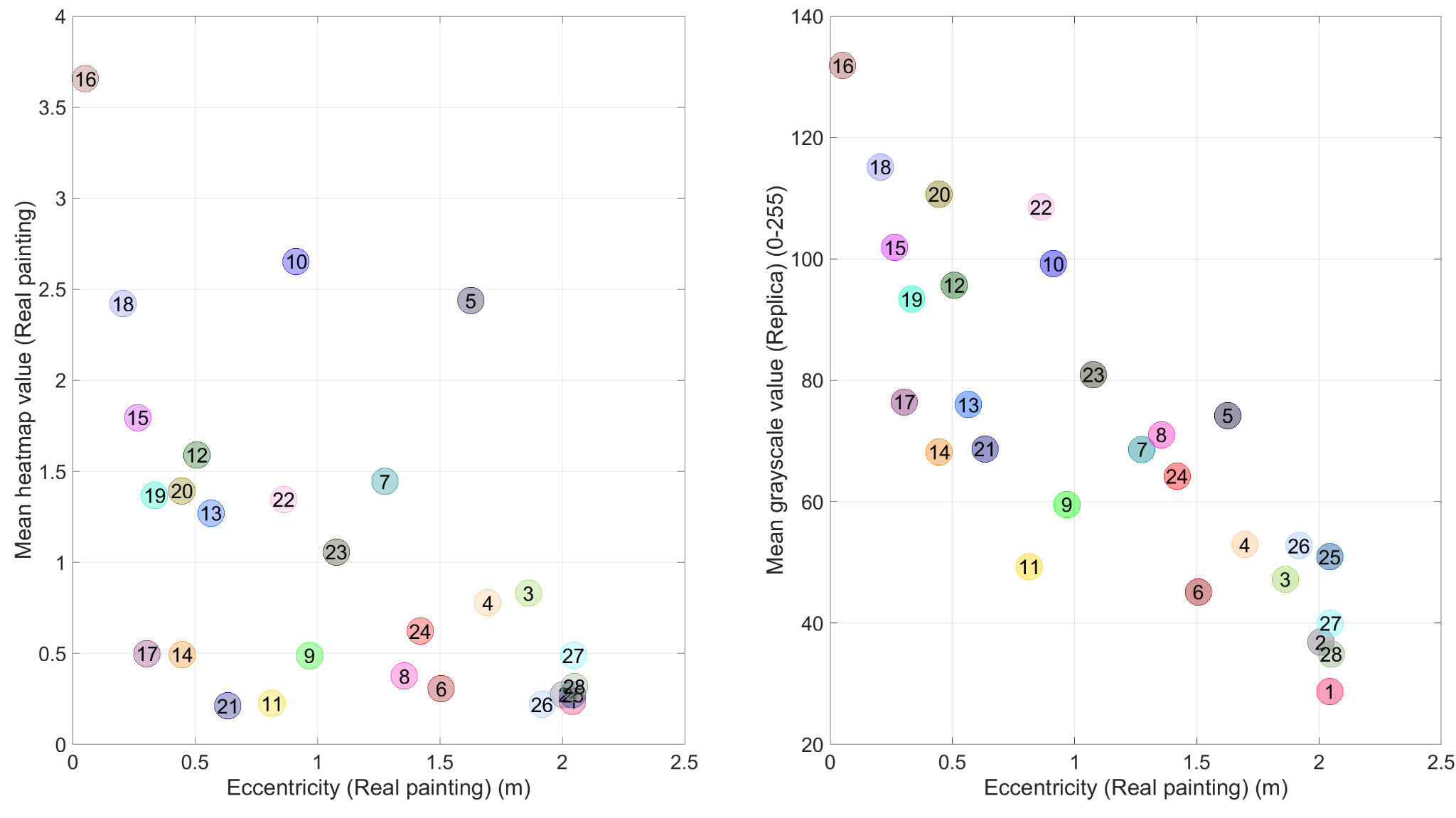


*Figure S1.* Left: Scatter plot of the mean heatmap value versus eccentricity for the real painting. Right: Scatter plot of the mean grayscale value of the replica versus eccentricity. The numbers correspond to the areas of interest shown in Figure 7.


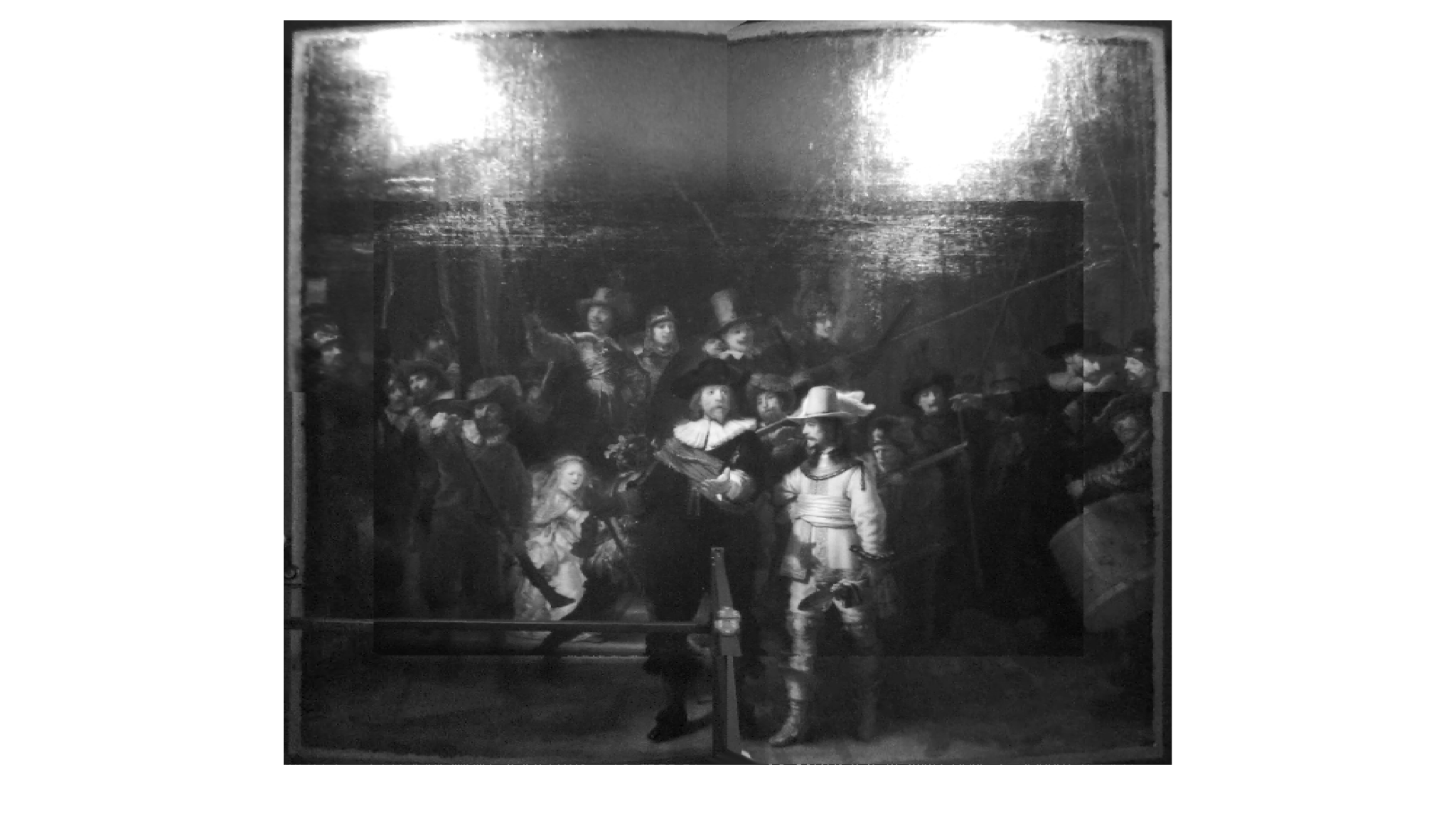


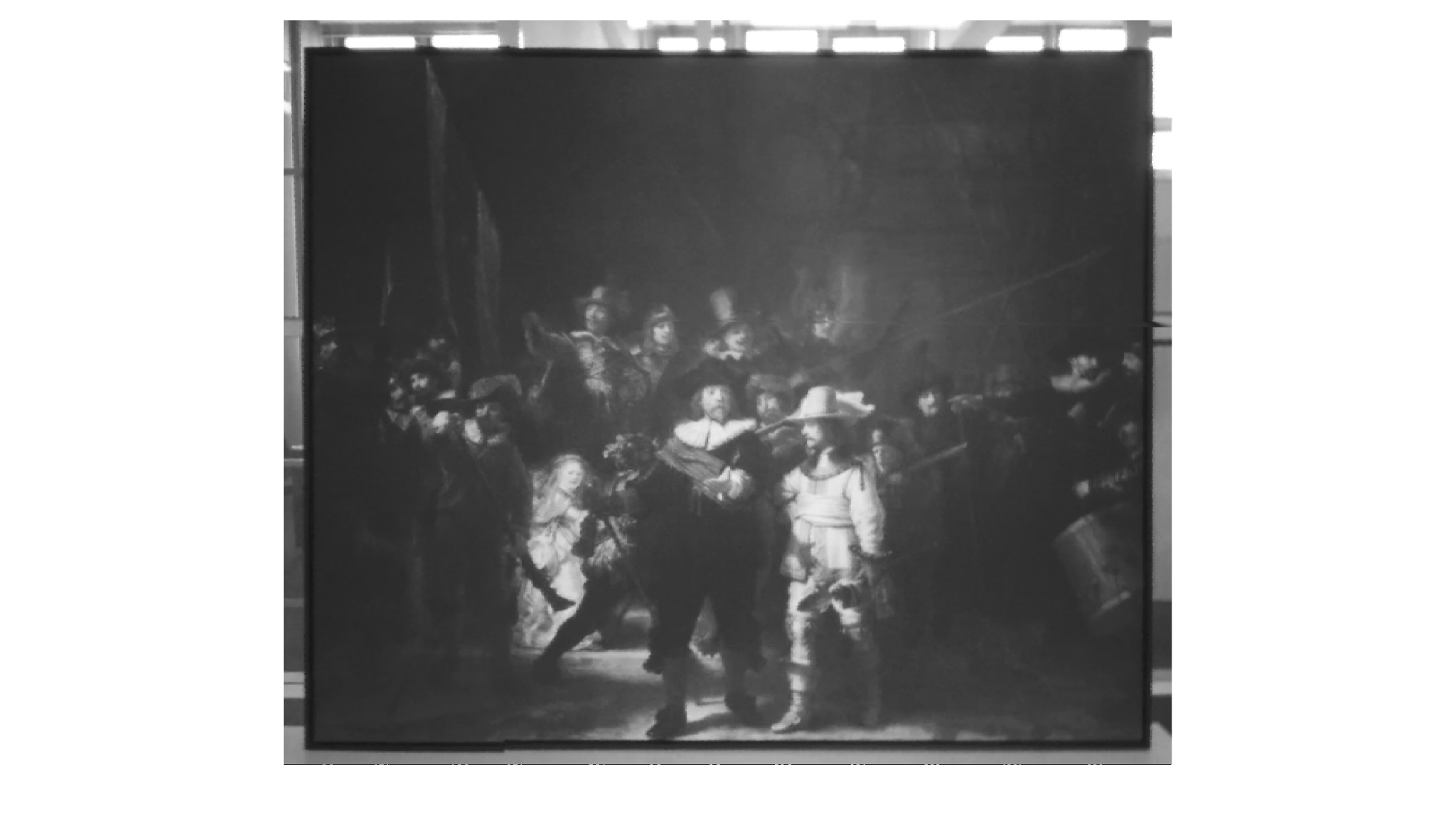
*Figure S2.* Top: Reconstructed frontal view from five reference images (see Figure 2) of the real *Night Watch*. Bottom: Reconstructed frontal view from a reference image of the replica *Night Watch* (bottom) (see Figure 8). The reconstructions were performed using Eqs. (1) and (2).


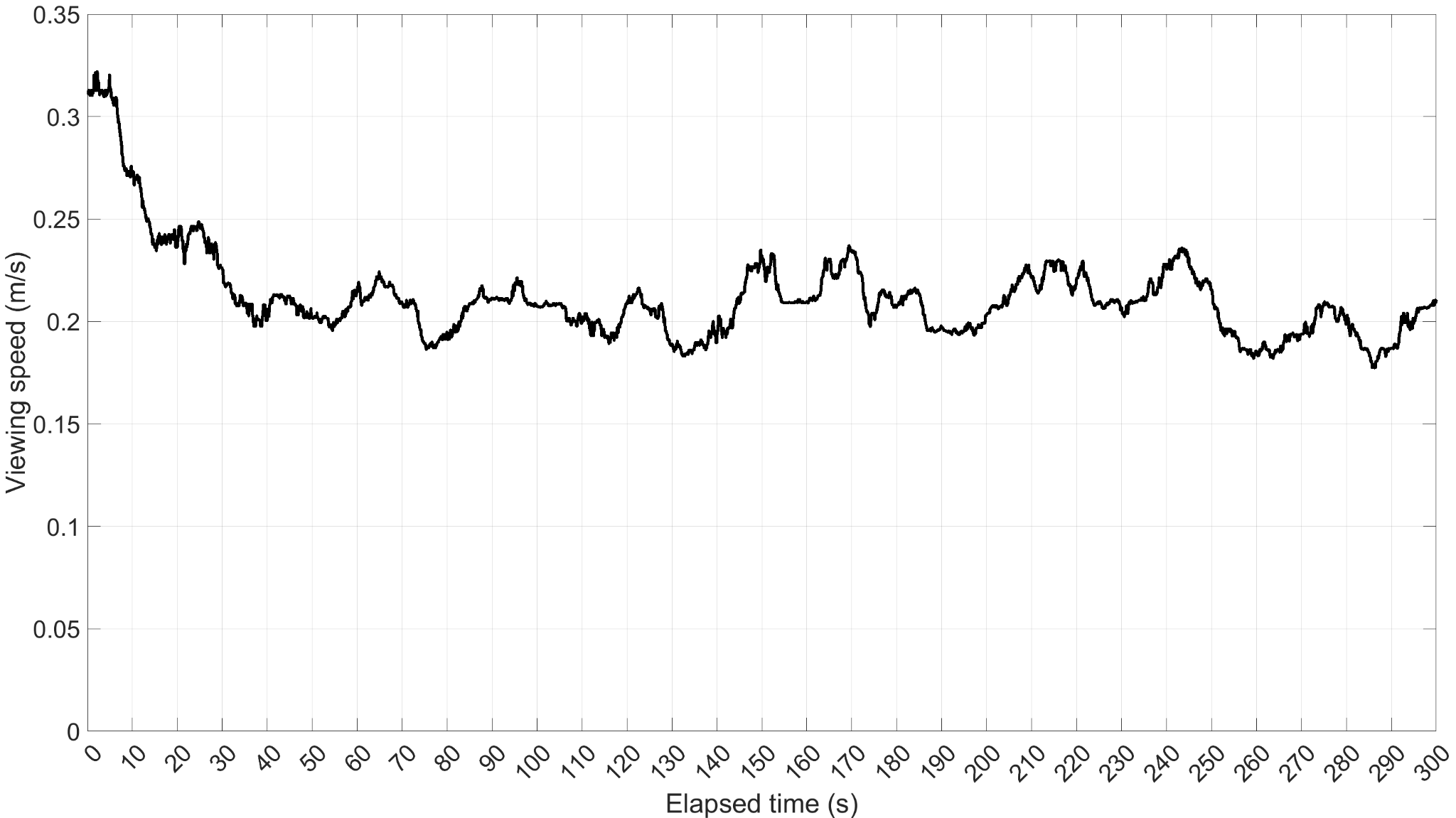


*Figure S3.* Viewing speed of the replica *Night Watch*, i.e., the speed of movement of the gaze point on the surface of the painting. This figure represents the median of all participants, which was subsequently filtered using a median filter with a window length of 250 (10 s).
